# A novel viral vaccine platform based on engineered transfer RNA

**DOI:** 10.1080/22221751.2022.2157339

**Published:** 2022-12-18

**Authors:** Tong-Yun Wang, Fan-Dan Meng, Guo-Ju Sang, Hong-Liang Zhang, Zhi-Jun Tian, Hao Zheng, Xue-Hui Cai, Yan-Dong Tang

**Affiliations:** a State Key Laboratory of Veterinary Biotechnology, Harbin Veterinary Research Institute of Chinese Academy of Agricultural Sciences, Harbin, People’s Republic of China; b Shanghai Veterinary Research Institute of Chinese Academy of Agricultural Sciences, Shanghai, People's Republic of China; c Heilongjiang Provincial Key Laboratory of Veterinary Immunology, Harbin, People's Republic of China; d Heilongjiang Provincial Research Center for Veterinary Biomedicine, Harbin, People's Republic of China

**Keywords:** Engineered transfer RNA, PTC virus, vaccine, switch, Pseudorabies virus, novel vaccine

## Abstract

In recent years, an increasing number of emerging and remerging virus outbreaks have occurred and the rapid development of vaccines against these viruses has been crucial. Controlling the replication of premature termination codon (PTC)-containing viruses is a promising approach to generate live but replication-defective viruses that can be used for potent vaccines. Here, we used anticodon-engineered transfer RNAs (ACE-tRNAs) as powerful precision switches to control the replication of PTC-containing viruses. We showed that ACE-tRNAs display higher potency of reading through PTCs than genetic code expansion (GCE) technology. Interestingly, ACE-tRNA has a site preference that may influence its read-through efficacy. We further attempted to use ACE-tRNAs as a novel viral vaccine platform. Using a human immunodeficiency virus type 1 (HIV-1) pseudotyped virus as an RNA virus model, we found that ACE-tRNAs display high potency for read-through viral PTCs and precisely control their production. Pseudorabies virus (PRV), a herpesvirus, was used as a DNA virus model. We found that ACE-tRNAs display high potency for reading through viral PTCs and precisely controlling PTC-containing virus replication. In addition, PTC-engineered PRV completely attenuated and lost virulence in mice *in vivo*, and immunization with PRV containing a PTC elicited a robust immune response and provided complete protection against wild-type PRV challenge. Overall, replication-controllable PTC-containing viruses based on ACE-tRNAs provide a new strategy to rapidly attenuate virus infection and prime robust immune responses. This technology can be used as a platform for rapidly developing viral vaccines in the future.

## Introduction

An increasing number of emerging virus outbreaks have occurred in humans over the past two decades, and some of them have caused substantial morbidity and mortality, such as severe acute respiratory syndrome coronavirus (SARS-CoV) [1,2], Middle East respiratory syndrome coronavirus (MERS-CoV) [3], Ebola virus [4], Zika virus [5] and severe acute respiratory syndrome coronavirus 2 (SARS-CoV-2) [6]. Vaccination is without question one of the most effective methods for preventing many viral diseases [7]. Traditional vaccines, including live attenuated viruses (LAVs) and killed or inactivated vaccines, always raise concerns; for example, LAVs pose several safety issues, and inactivated viruses are generally less efficacious than their LAV counterparts [7,8]. The classical attenuation process of LAVs is somewhat unpredictable and has not always been applicable. The outbreak of COVID-19 will almost certainly change the future of vaccine science, and the vaccine development process must be accelerated to guarantee safety [9]. With the development of synthetic biology, novel biotechnologies have been applied in vaccinology [10,11]. Genetic code expansion (GCE) technology is a promising approach to generate live but replication-incompetent viruses that are used as an ideal vaccine, avoiding the unpredictability of empirical attenuation [12]. Genetic code expansion technology involves an orthogonal translation system that precisely controls the replication of viruses harbouring premature termination codons (PTCs) through the cooperation of the *Methanosarcina barkeri* Mb pyrrolysyl tRNA synthetase/tRNA^CUA^ pair (MbpylRS/tRNA^CUA^) with an orthogonal unnatural amino acid (UAA) [12]. Hepatitis D virus [13], HIV-1 [14,15], Zika virus [16] and pseudorabies virus[17] replication have been reported to be precisely controlled by GCE technology. A potent vaccine against influenza virus has been successfully developed using GCE, and it elicited robust immunity against both parental and antigenically distinct strains [12]. The decisive, key step of GCE technology is the efficacy of UAA incorporation into the desired PTC engineering sites; however, it seemed extremely low in some tested viruses, which may limit the broad application of this technology [16,17].

**Figure 1.**
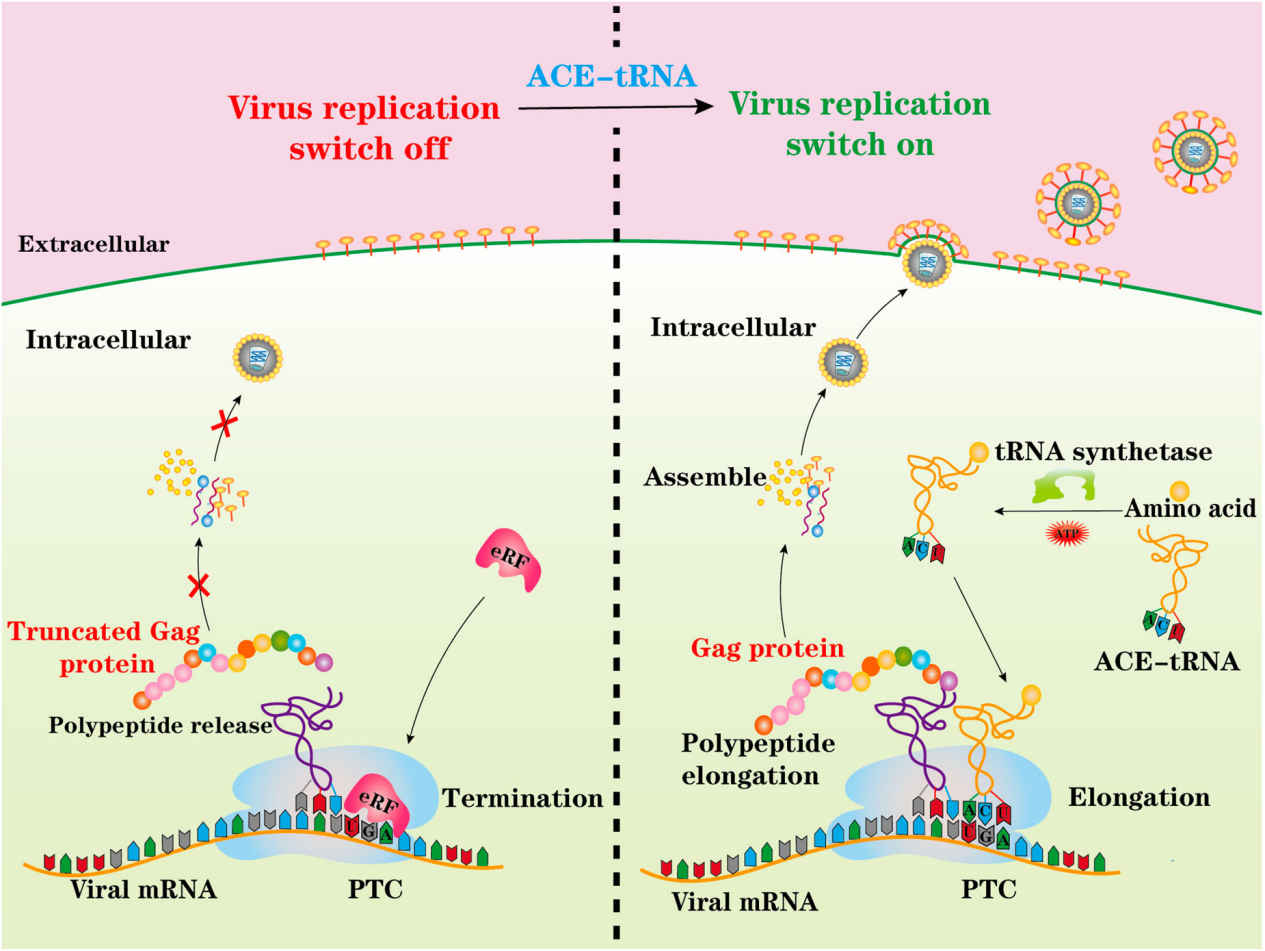
Schematic illustrating the principal of this study. A nonsense mutation PTC-containing virus was engineered and rescued by ACE-tRNAs. ACE-tRNAs are recognized by an endogenous aminoacyl-tRNA synthetase charged with their cognate amino acid. Aminoacylated ACE-tRNA is recognized by endogenous elongation factor 1-alpha (EF1α) and delivers the aminoacyl ACE-tRNA to the ribosome for PTC read-through, achieving PTC-harbouring virus rescue (right panel). In the absence of ACE-tRNAs, PTCs are recognized by eukaryotic release factor (eRF), and polypeptide elongation is terminated (left panel).

In the present study, we used anticodon-engineered transfer RNAs (ACE-tRNAs) as an alternative promising PTC read-through tool to efficiently generate a PTC-containing virus. Unlike GCE technology, ACE-tRNAs are recognized by endogenous aminoacyl-tRNA synthetase to charge ACE-tRNAs with their natural cognate amino acid to read-through PTCs [18]. Therefore, ACE-tRNAs and their application to PTC viruses may provide a new strategy to generate live but replication-incompetent viruses, as illustrated in Figure 1. We systemically investigated the potential PTC read-through ability of three representative ACE-tRNAs, and we showed that ACE-tRNAs display higher potency of reading through PTCs than GCE technology. Furthermore, in this study, we used an HIV-1 pseudotyped virus as a model RNA virus to generate an HIV-1-PTC virus and showed that its replication was precisely controlled by ACE-tRNAs. Pseudorabies virus (PRV), a herpesvirus, was used as a model DNA virus, and two PRV-PTC viruses were generated to test the possibility of applying ACE-tRNAs in vaccine development. We showed that either PRV-PTC virus completely lost virulence in mice *in vivo*. Immunization with PRV-PTC virus elicited a robust immune response and provided potent protection against challenge with wild-type PRV. Overall, ACE-tRNAs may represent a promising approach for the rapid development of vaccines and may be broadly applied to other viruses.

## Materials and methods

### Cells and plasmids

Human embryonic kidney 293T (HEK-293T, ATCC CRL-11268) cells, rabbit kidney cells (RK13, ATCC CCL-37), and Vero cells (ATCC CCL-81) were maintained in DMEM (Gibco, USA) supplemented with 10% (v/v) fetal bovine serum (Excell, Australia). The PRV JX-2012 BAC plasmid was as described in our previous report [19]. pDC315-EGFP is an EGFP expression plasmid described in our previous studies [20,21]. The pLVX-IRES-ZSGreen (Addgene, USA) or HIV-1-based luciferase (HIV-Luc) reporter plasmid [20,22] was cotransfected with the helper plasmids psPAX2 and pMD2.G (Addgene, USA). The MbPylRS/tRNA_CUA_ plasmid was described in our recent study [17].

tRNA gene sequences were obtained from the tRNA database (http://gtrnadb.ucsc.edu/index.html) [18]. Three ACE-tRNA (ACE-Arg^UGA^, ACE-Gly^UGA^ and ACE-Trp^UGA^) expression plasmids were synthesized directly by Sangon Biotech Company (Shanghai, China) and cloned into the pUC57 vector. These ACE-tRNAs contain 4 tandem tRNAs driven by different eukaryotic promotors.

### Construction of PTC mutants of pDC315-EGFP

Glycines at positions 5, 11, 21, 25, 32, 34, 36, 41, 52, 68, 92, 105, 117, 128, 135, 175, and 190, tryptophan at position 58, arginines at positions 74, 110, 123, and 169, and lysines at positions 4, 27, 42, 53, 80, 86, 114, 127, 132, 159, 163, 167 in EGFP were mutated to the opal stop codons TGA and TAG, respectively. Briefly, the codon of the indicated amino acid was replaced by a TGA stop codon through site-directed mutagenesis using PCR. All clones were verified by DNA sequencing. The primers used for site-directed mutagenesis are listed in Tables S1 and S2.

### Read through of EGFP PTC by ACE-tRNA

The opal (UGA) translation stop codon was introduced into the EGFP gene, and the indicated ACE-tRNA was cotransfected into HEK293T cells. Briefly, HEK293T cells were plated in 24-well plates (4 × 10^5^ cells/well), and 1 µg of the indicated ACE-tRNA (Trp-tRNA^UGA^/Arg-tRNA^UGA^/Gly-tRNA^UGA^) or control vector was cotransfected with 1 µg of pDC315-EGFP PTC plasmid using jetPRIME (Polyplus, France). pDC315-EGFP was used as a positive control, and nontransfected 293T cells were used as mock cells. At 6 h posttransfection, the supernatant was replaced with DMEM supplemented with 2% FBS. Fluorescence microscopy and flow cytometry were used to analyse the read-through efficacy of ACE-tRNA.

### Construction of the psPAPX2 plasmid harbouring a single *SpeI* site

Two *SpeI* restriction sites are present in psPAPX2. We first removed one *SpeI* site using PCR to facilitate our experiment. Briefly, a point mutation was introduced in the forward primer: 5’-GACATTGATTATTG*GCTAGT*TATTAATAGTAATC-3’; the reverse primer was 5’-CCTATTTGTTCCTGAAGGGTAC-3’. Homologous recombination of the PCR products was conducted with a *SpeI*-predigested psPAPX2 and ClonExpress II One Step Cloning Kit (Vazyme, China). The newly constructed plasmid was named psPAPX2-mu-*SpeI*.

PTC plasmids containing one PTC (Gly-221-PTC, Gly-226-PTC, Arg-229-PTC, Arg-232-PTC, Gly-233-PTC, or Gly-238-PTC) and two PTCs (Gly-221-226-PTC, Gly-221-238-PTC, or Arg-229-232-PTC) in the HIV-1 gag region were generated from psPAPX2-mu-*SpeI* via fragment substitution between *SpeI* and *SphI*. First, oligonucleotides with the TGA mutation sites mentioned above were synthesized directly by Comate Bioscience in China and then assembled into *SpeI-* and *SphI*-predigested psPAPX2-mu-*SpeI*. All clones were confirmed by DNA sequencing. Oligonucleotides used for the construction of single and double PTCs of the gag gene are listed in Table S3.

### PTC lentiviral packaging

PLVX-IRES-ZSGreen or HIV-Luc was cotransfected with the helper plasmid pMD2.G and the indicated PTC psPAPX2 plasmids (PTC-Gly-221, PTC-Gly-226, PTC-Arg-229, PTC-Arg-232, PTC-Gly-233, and PTC-Gly-238) as previously described [20,22]. HEK293T cells were used for lentiviral vector packaging and infectivity titration. HEK293T cells (2 × 10^5^ cells/well) were plated in 6-well plates in DMEM supplemented with 10% FBS. The cells were cotransfected with 0.5 µg of pMD2. G, 1 µg of psPAPX2 or the PTC plasmid in psPAPX2, 1.5 µg PLVX-IRES-ZSGreen or HIV-Luc as well as 1 µg ACE-tRNA (Trp-tRNA^UGA^/Arg-tRNA^UGA^/Gly-tRNA^UGA^) or the empty vector pUC57 using the jetPRIME transform reagent. At 6 h posttransfection, the cell culture medium was replaced with DMEM supplemented with 10% FBS. Next, the supernatant containing the lentivirus was harvested at 48 h posttransfection and centrifuged at 1000 × g for 5 min, followed by filtration through a 0.45-µm MCE-membrane syringe filter (Millipore, USA) to remove the cell debris. The packaged lentivirus was stored at −80°C.

### Detection of the ACE-tRNA suppression efficacy of the PTC-containing virus using Western blotting

PTC-containing viruses were produced as described above. The PTC-containing virus in the supernatant was collected at 48 h posttransfection and centrifuged at 10,000 × g for 10 min to remove the cell debris; virions were centrifuged at 20,000 rpm at 4°C for 2 h for pelleting. The viral pellets and PTC-containing virus in cells were subjected to Western blot analysis as previously described [23].

### Assay of PTC-containing viral infection

HEK293T cells in a 24-well plate were inoculated with 200 µL of the PTC-containing virus and incubated at 37°C for 3 h for viral absorption and entry. Next, the cells were washed with PBS to remove the unabsorbed virus, and DMEM supplemented with 2% FBS was added. Each viral infection was repeated three times. At 48 hpi, the cells were treated as described below. For the EGFP reporter assay of PTC-containing virus infection, fluorescence microscopy and flow cytometry were used to analyse the infectivity of the PTC-containing virus. For fluorescence microscopy, the infected cells were fixed with 4% paraformaldehyde for 40 min and then washed with PBS three times. For nuclear visualization, the cells were stained with DAPI (Sigma, USA). For flow cytometry experiments, the infected cells were digested directly with trypsin and analysed using flow cytometry.

For the luciferase reporter assay of PTC-harbouring virus infectivity, HEK293T cells growing in a 48-well cell plate were inoculated with 200 µL of PTC-containing virus per well; at 48 hpi, the cells were lysed with 1× Reporter Lysis Buffer. A Promega E1500 Luciferase Assay System kit (Promega, USA) was used to detect luciferase activity.

### Comparison of the read-through efficacy for PTC-harbouring EGFP using GCE and ACE-tRNA technology

The amber (UAG) and opal (UGA) stop codons were introduced into the EGFP gene by using site-directed mutagenesis and PCR. HEK-293T cells were seeded in 24-well plates (2 × 10^5^ cells/well) prior to transfection. For GCE technology, 0.2 μg of the pDC315-EGFP-TAG PTC plasmid or pDC315-EGFP wild-type plasmid was cotransfected into HEK-293T cells with 0.2 μg of the MbPylRS/tRNA_CUA_ plasmid using jetPRIME. Two parallel experiments were conducted. The culture medium was replaced with fresh medium containing 2% FBS with or without 1 mM UAA (NAEK) supplement at 6 h posttransfection.

For ACE-tRNA technology, 0.2 µg of the indicated ACE-tRNA (Trp-tRNA^UGA^/Arg-tRNA^UGA^/Gly-tRNA^UGA^) or control vector was cotransfected with 0.2 µg of the pDC315-EGFP PTC plasmid. The pDC315-EGFP-transfected group was used as a positive control, and the mock-transfected group was used as a negative control. The PTC read-through efficacy was detected by performing fluorescence microscopy and flow cytometry at 48 h posttransfection.

### Anti-codon editing for the orthogonal tRNA used in GCE technology

Plasmids containing four tandem indicated tRNA^UCA^ were synthesized by Sangon Biotech Company (Shanghai, China). Two sets of orthogonal translation systems containing tRNA^CUA^ and tRNA^UCA^ were compared, in which the same aminoacyl tRNA synthase Mbpy was used. A total of 0.3 µg of the pDC315-EGFP-PTC plasmid, 0.15 µg of MbpylRS and 0.15 µg of tRNA^CUA^ were cotransfected into HEK-293T cells in 48-well plates, and 0.3 µg of the pDC315-EGFP PTC plasmid was cotransfected with 0.15 µg of MbpylRS and 0.15 µg of tRNA^CUA^. Two parallel experiments were conducted. The culture medium was replaced with fresh medium containing 2% FBS with or without 1 mM UAA (NAEK) supplement at 6 h posttransfection. The PTC read-through efficacy was detected by performing fluorescence microscopy and flow cytometry at 48 h posttransfection.

### Construction of the gB plasmid harbouring PTC

Arginines at positions 9, 26, 63, 111, 115, 123, 137, 151, 196, 226, 246, 256, 290, 315, 329, 357 and glycines at positions 17, 39, 57, 79, 91, 132, 187, 216, 254, 303, 328, 394, 404, 422 of the PRV *gB* open reading frame (ORF) were replaced with an opal stop codon (UGA) via site-directed mutagenesis and PCR. The related primers are listed in Table S4. PTC-containing mutant gB constructs were confirmed by DNA sequencing (Rui Biotech, China).

#### Analysis of the read-through efficacy for gB PTC induced by ACE-tRNA using a cell-to-cell fusion assay

Cell-to-cell fusion assays were performed as previously described [24–26]. RK13 cells were seeded in 24-well plates. Then, 150 ng of plasmids encoding *gB* or its mutants were cotransfected into RK13 cells with gD, gH, gL, and pDC315-EGFP and 400 ng of the indicated ACE-tRNA (Arg-tRNA^UGA^/Gly-tRNA^UGA^). pUC57 was used as the control group. Nontransfected RK13 cells were used as a mock control. The culture medium was replaced with fresh medium supplemented with 2% FBS at 6 h posttransfection. The read-through efficacy of ACE-tRNA was detected by analysing gB-mediated cell-to-cell fusion.

#### Western blot analysis of the read-through efficacy of gB PTC induced by ACE-tRNA

HEK-293T cells exhibiting good growth were plated in 24-well plates (4 × 10^5^ cells/well). When the cell confluence reached 80%, 1 µg of the indicated ACE-tRNA (Arg-tRNA^UGA^ or Gly-tRNA^UGA^) plasmid was cotransfected with 1 µg of the corresponding *gB* PTC plasmid. The pUC57 control and wild-type *gB* plasmid groups were used as the negative control and positive control, respectively. At 48 h posttransfection, Western blotting was performed to analyse the read-through efficacy of gB harbouring PTC mutants induced by ACE-tRNA.

#### Construction of PRV PTC-gB

The PRV-PTC BAC infectious clone was constructed as previously described [17,19]. The obtained BAC clone with the TGA PTC in gB was termed PTC-pPRV-Bac-JS2012.

### Rescue of PRV-PTC

HEK-293T cells and Vero cells were plated in 6-well plates in DMEM supplemented with 10% FBS. Two micrograms of Arg-tRNA^UGA^ were cotransfected with 2 µg of the indicated PTC-pPRV-Bac-JS2012 (pPRV-Arg-151-PTC or pPRV-Arg-196-PTC) using jetPRIME. A parallel experiment was conducted by cotransfecting cells with the empty vector pUC57 and the indicated pPRV-PTC-Bac, which served as a negative control, to determine the Arg-tRNA^UGA^ dependence of PRV-PTC. Nontransfected HEK-293T cells were used as mock controls. The culture medium was replaced with DMEM containing 2% FBS at 6 h posttransfection. The cells were further incubated at 37°C with 5% CO_2_ until a cytopathic effect (CPE) or syncytium was observed.

### Evaluation of the genetic stability of PRV-PTC

HEK-293T cells were used for PRV-PTC amplification and infectivity assays. Eighty percent confluent HEK-293T cells were transfected with 3 µg of Arg-tRNA^UGA^ or the empty vector pUC57. Twenty-four hours after transfection, the rescued PTC-containing virus was collected and centrifuged at 1000 × g for 5 min, followed by filtration through a 0.45-µm filter to remove the cell debris before a new round of infection in DMEM supplemented with 2% FBS. The PTC-containing virus was passaged 3 times in the presence of Arg-tRNA^UGA^. A parallel experiment in which the cells were transfected with pUC57 was conducted as a negative control.

After each passage, viral DNA was extracted from 200 µL of the cell supernatant mixture using the Virus Genome DNA/RNA Extraction Kit (Tiangen, Beijing) according to the manufacturer’s procedure. The PTC-gB fragment of PRV-PTC was amplified by PCR followed by genome sequencing to detect the genetic stability of PRV-PTC virus during viral passaging. The primers used for PCR and sequencing are as follows: F: CGTCTTTGGCCTGCTCCACACCACG; R: CGCCGATCTTGGTGTAGGTGTCGTTG.

#### Analysis of virus growth properties

HEK-293T cells (2 × 10^6^ cells/well) were seeded in 6-well plates, and 2 µg of Arg-tRNA^UGA^ were cotransfected with 2 µg of the indicated PTC-pPRV-Bac-JS2012 (pPRV-Arg-151-PTC or pPRV-Arg-196-PTC) to determine the virus growth capacity *in vitro*. Two micrograms of pUC57 and 2 µg of pPRV-Bac-JS2012 were cotransfected as positive controls. At the indicated times after transfection (24, 36, 48 and 72 h), the PTC-containing virus in cells and supernatants was collected, and the viral titres were determined by calculating the TCID_50_.

### Transmission electron microscopy of PRV-PTC

HEK-293T cells were transfected with pPRV-Bac-JS2012 or PTC-pPRV-Bac-JS2012 in the presence or absence of Arg-tRNA^UGA^ and then fixed with 2.5% (w/v) glutaraldehyde in 200 mM HEPES (pH 7.4) for 2 h at room temperature. The samples were postfixed with 1% OsO_4_ and 1.5% K_3_Fe(CN)_6_ in H_2_O at 4°C for 30 min. The samples were dehydrated with acetone, impregnated with epoxy at room temperature and subsequently embedded overnight at 70°C for polymerization using standard procedures. Then, the samples were cut into 70 nm ultrathin sections with an ultrathin slicer (Leica, Germany) and stained with 2% uranium acetate for 17 min and lead citrate for 12 min. The specimens were examined under a conventional transmission electron microscope (TEM, h7650, Hitachi, Japan).

#### RT–qPCR for detecting mRNA expression

HEK-293T cells were seeded in 6-well plates. Upon reaching 80% confluence, the cells were transfected with 2 μg of the indicated plasmids (GFP-PTC or GFP-WT and Gag-PTC or Gag-WT). Total cellular RNA was extracted from the transfected cells at 36 h posttransfection using an RNeasy® Plus Mini Kit (QIAGEN, China). In addition, 2 U of RNase-free recombinant *DNase I* (BioLabs, USA) were incubated with the samples at 37°C for 30 min to eliminate the effect of transfected plasmid DNA, followed by the addition of 0.5 M EDTA at 75°C for 10 min to inactivate *DNase I*. RT–qPCR was performed to quantify the mRNA levels of GFP-PTC and Gag-PTC mutants. One microgram of RNA was reverse transcribed into cDNAs using HiScript Reverse Transcriptase (Vazyme Biotech, China). The related primers and probes used for the RT–qPCR analysis are listed in Table S5. Data were analysed using the MxPro-Mx3005P (standalone) RT–qPCR system.

### Animal experiments

#### Preparation of inactivated PRV

The inactivated virus was prepared by mixing β-propanolactone (Sigma–Aldrich, USA) and wild-type PRV (1 × 10^5^ TCID_50_/mL) at a ratio of 1:2000 and incubating them at 4°C for 48 h with mixing of the mixture several times during the incubation period. Then, the mixture was homogenized with the mineral MONTANIDE™ ISA 206 (SEPPIC, France), a well-known adjuvant, in a W/O/W emulsion.

#### Evaluation of the safety of PRV-PTC in mice

To evaluate the safety of PRV-PTC virus, Female specific-pathogen free (SPF) BALB/c mice aged 6–10 weeks old were used to evaluate the safety of PRV-PTC and randomly divided into 6 groups (7 mice per group). Six groups of mice were inoculated with 10^4^ TCID_50_ wild-type PRV Bac-JS2012 virus, 10^4^ TCID_50_ PRV-Arg-151-PTC virus, 10^4^ TCID_50_ PRV-Arg-196-PTC virus, 10^5^ TCID_50_ PRV-Arg-151-PTC virus, 10^5^ TCID_50_ PRV-Arg-196-PTC virus, or DMEM as a control (all via subcutaneous inguinal injection). One mouse from each group was sacrificed on day 4 postinoculation, and their brains were collected for hematoxylin and eosin (H&E) staining. The remaining six mice were observed daily for three weeks to determine the survival rate.

#### Protective efficacy of PRV-PTC

SPF BALB/c mice aged 6–10 weeks old were used to evaluate the protective efficacy of PRV-PTC and randomly divided into 5 groups (6 mice per group). Groups of five mice were subcutaneously and inguinally injected twice with 10^5^ TCID_50_ PRV-Arg-151-PTC virus, 10^5^ TCID_50_ PRV-Arg-196-PTC virus, 10^5^ inactive vaccine or DMEM as a control, with three weeks between the two immunizations. On the 21st day after the second immunization, BALB/c mice were challenged with 3 × 10^4^ TCID_50_ of the highly virulent PRV strain Bac-JS2012 via subcutaneous inguinal injection in a total volume of 0.1 mL. The animal survival rate and clinical symptoms were monitored for 14 days.

#### Virus-neutralizing antibody detection

Serum neutralizing antibodies against PRV were determined by performing a neutralization assay. The complement in sera was inactivated by heating at 56°C for 30 min, and then two-fold serial dilutions of the sera were incubated with the PRV JS2012 strain (50 TCID_50_) for 2 h at 37°C. Subsequently, the mixtures were added to Vero cells, and the results of the serum neutralizing antibody titre are presented as the highest serum dilution that completely inhibited the CPE.

#### Cytokine production in mice

The serum was separated and collected from challenged mice at 24 hpi, and cytokine production was analysed using ELISA. The concentrations of IFN-γ and TNF-α in mouse serum were determined using commercial mouse IFN-γ and TNFα immunoassay kits according to the manufacturer’s instructions (Wuhan Huamei Bio-Technology Co., CUSABIO, China). Supplied standards were used to generate a standard curve. The detection limits of the assays were 2.0 pg/mL for IFN-γ and 62.5 pg/mL for TNF-α.

#### H&E histopathological analyses and determination of the virus load in tissues

On the 14th day after challenge, the liver, heart, spleen, brain, lung and kidney were collected (six mice per group) for viral load detection and H&E staining and histopathological analyses. For viral load detection, the total viral DNA was extracted from 25 mg tissue samples using a MagaBio plus Virus DNA/RNA Extraction Kit (BIOFLUX; Hangzhou, China) according to the manufacturer’s instructions. Viral copies were determined using RT–qPCR. The probe and primers are listed in Table S5.

#### Statistical analysis

GraphPad Prism 9.0 software was used to prepare all graphical representations. Statistical significance was analysed using one-way ANOVA and Tukey’s multiple comparison test or the independent Student’s t test. A *p* value < 0.05 was considered statistically significant.

## Results

### Evaluation of the PTC read-through activity of ACE-tRNAs

**Figure 2.**
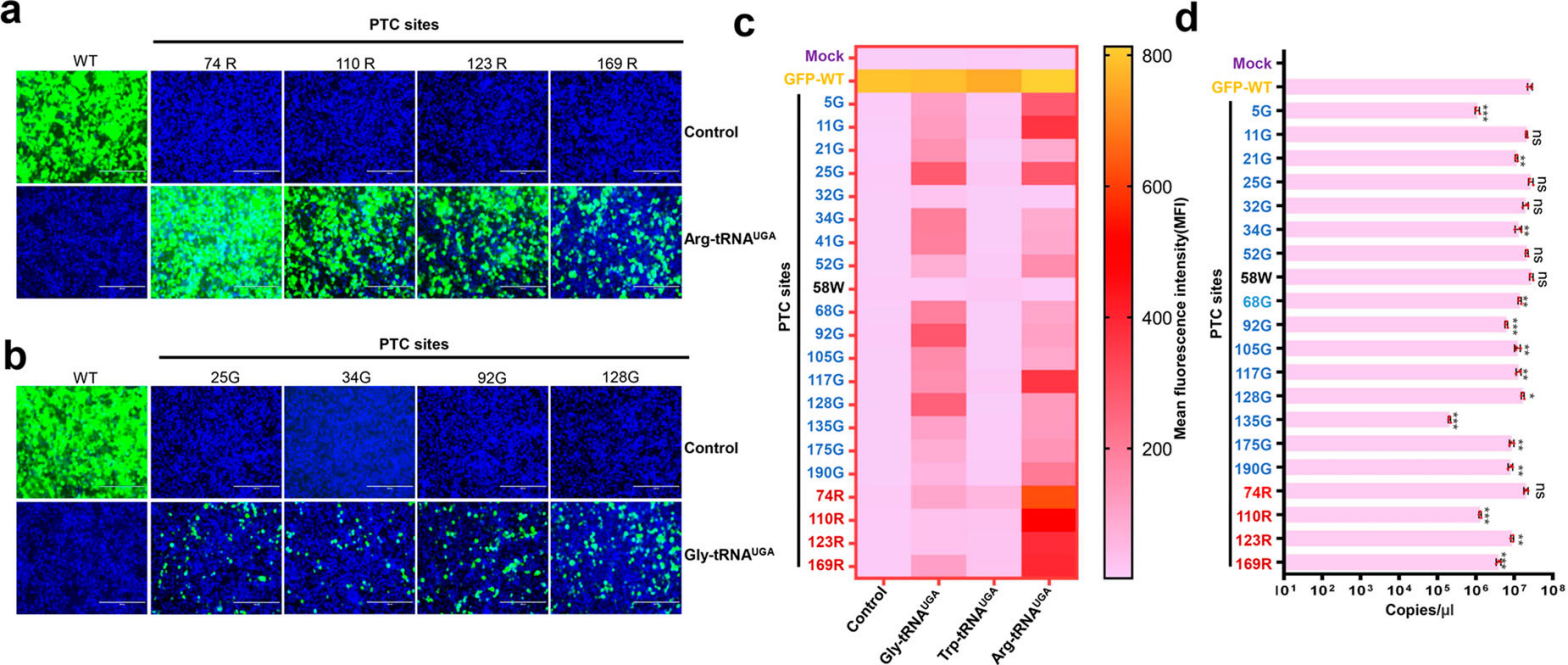
ACE-tRNA manifested potent PTC read-through activity. (a) Arg-tRNA^UGA^ and (b) Gly-tRNA^UGA^ were cotransfected with the indicated PTC EGFP in HEK293T cells. At 48 h posttransfection, fluorescence microscopy was used to evaluate the ACE-tRNA suppression efficacy. The wild-type EGFP group was used as a positive control, and the nontransfected group was used as a negative control. Cell nuclei were stained with DAPI; the scale bar represents 200 μm. (c) Heatmap of the EGFP of PTC suppression efficacy mediated by the three ACE-tRNAs. The MFI of EGFP was evaluated using flow cytometry. The nontransfected group was used as a mock control, and the pDC315-EGFP PTC plasmid and ACE-tRNA empty vector pUC57 cotransfected groups were used as negative controls. Experiments were repeated three times, and the mean MFI is presented. (d) Detection of the mRNA levels of PTC-EGFP mutants using RT–qPCR. Experiments were repeated three times.

We first investigated the PTC read-through activity of three representative ACE-tRNAs: Arg-tRNA^UGA^, Gly-tRNA^UGA^ and Trp-tRNA^UGA^ [18]. The PTC of the opal codon (UGA) was engineered into the EGFP coding region, including 1 tryptophan, 4 arginines and 17 glycines. Next, we cotransfected the PTC-EGFP plasmid together with the indicated ACE-tRNA plasmid or vector into HEK293T cells. As expected, no detectable fluorescence signal was observed for the PTC-EGFP constructs. However, PTC-EGFP cotransfection with ACE-tRNA restored the fluorescence signal to different levels. Notably, Arg-tRNA^UGA^ and Gly-tRNA^UGA^ exhibited more potent PTC read-through activity. All 4 PTCs carrying arginine at positions 74, 110, 123, and 169 in EGFP exhibited good read-through to varying extents (Figure 2(a)). Furthermore, the 17 PTCs carrying glycine were also read through by Gly-tRNA^UGA^; however, its read-through activity was lower than that of Arg-tRNA^UGA^ (Figure 2(b)). Only 1 tryptophan is present in the EGFP coding region, and we did not observe Trp-tRNA^UGA^ read-through activity at position 58 (Figure S1c).

We next examined whether these ACE-tRNAs displayed substitutability; interestingly, Arg-tRNA^UGA^ showed potent PTC read-through activity at Gly-PTCs (Figure S1a). Gly-tRNA^UGA^ also read-through Arg-PTCs at a low level (Figure S1b). In contrast, no EGFP signal was detectable for any PTC with Trp-tRNA^UGA^ (Figures S1c and S1d). Based on these results, Trp-tRNA^UGA^ may not be an ideal ACE-tRNA. Finally, we further quantified the read-through activity of these PTC-EGFPs using flow cytometry, and the read-through activities of all PTCs were evaluated by calculating the mean fluorescence intensity (MFI) (Figure 2(c)). A heatmap of the MFI indicated that ACE-tRNA may display a PTC site preference, with Arg-tRNA^UGA^ performing the best in this system. Importantly, mRNAs harbouring PTCs are selectively degraded by a nonsense-mediated mRNA decay (NMD) mechanism [27]. Thus, mRNA transcripts from PTC-EGFP constructs may be degraded by NMD. Next, we detected the mRNA levels of these PTC-EGFPs and found that the mRNA levels of some PTC-EGFP constructs were significantly decreased (Figure 2(d)). However, in some PTC-Glys, no difference was detected in mRNA levels, but PTC-25-Gly showed a higher read-through activity than other PTC-Gly sites (Figure 2(c,d)). This finding indicated that NMD and the PTC site preference collaboratively contributed to the PTC read-through activity of ACE-tRNAs.

### Comparison of the PTC read-through activity of GCE and ACE-tRNAs

**Figure 3.**
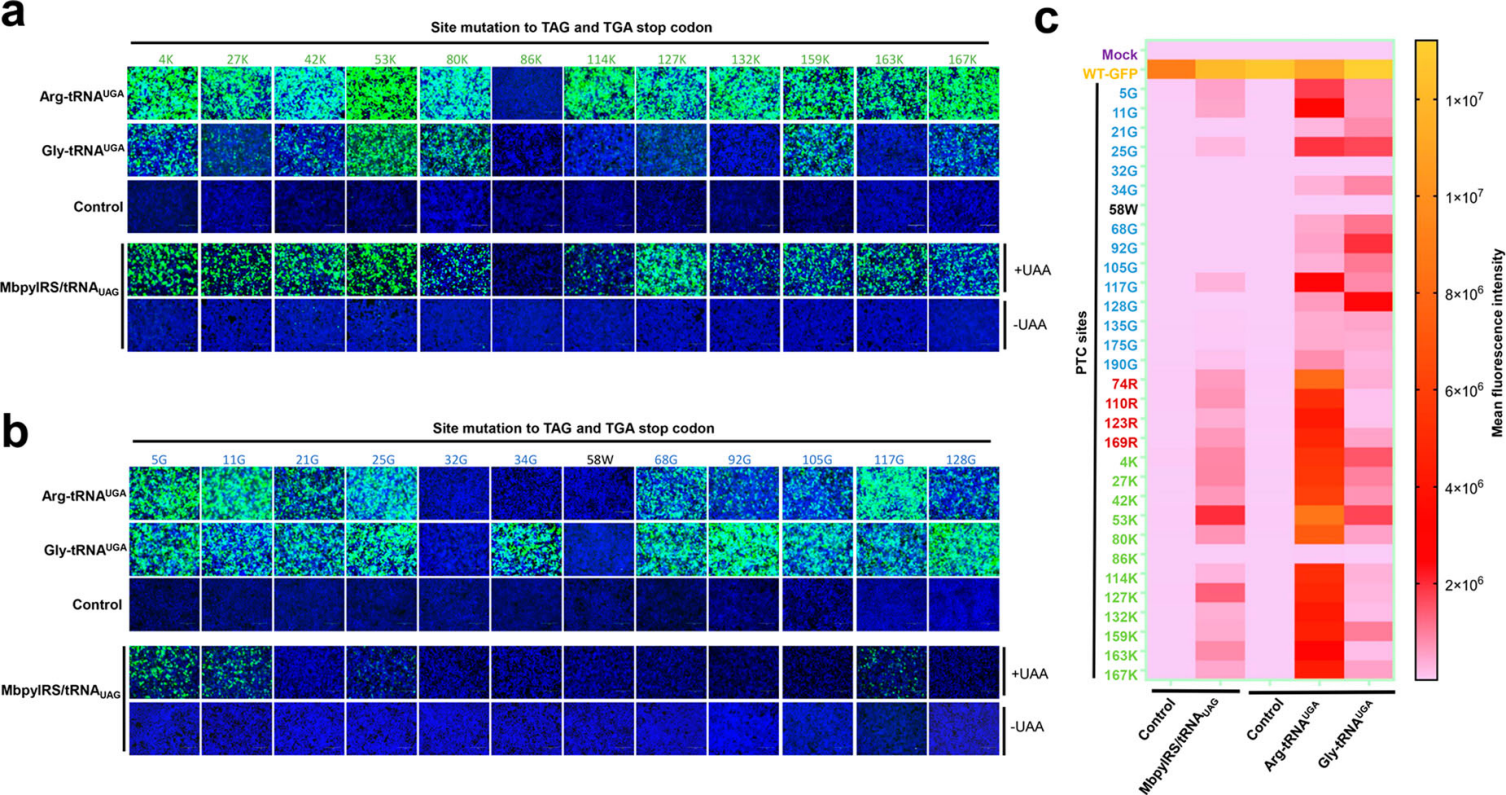
Comparison of the read-through efficacy of PTC sites using GCE and ACE-tRNA technology. (a) Arg-tRNA^UGA^, Gly-tRNA^UGA^ or control vector pUC57 was cotransfected with PTC-EGFP-Lys mutants or (b) PTC-EGFP-Gly mutants. The EGFP-WT-transfected group was used as a positive control, and the mock-transfected group was used as a negative control (upper panel). The EGFP-WT or PTC-EGFP plasmid was cotransfected with the MbPylRS/tRNA_CUA_ plasmid into HEK-293T cells. Two parallel experiments were conducted. Cell culture medium was replaced with fresh medium supplemented with 2% FBS with or without 1 mM UAA (NAEK) at 6 h posttransfection (lower panel). Fluorescence microscopy was used to compare the read-through efficacy of GCE and ACE-tRNA technology. (c) Heatmap of the PTC-EGFP read-through efficacy mediated by GCE and ACE-tRNA technology. The MFI of EGFP was evaluated using FACS. Experiments were repeated three times.

Next, we compared the PTC read-through efficacy of GCE and ACE-tRNA. Because GCE recognizes the amber codon (UAG), we further introduced the amber codon at the position of the opal codon. In the current study, we used the orthogonal translation system MbpylRS/tRNA^Pyl^ pair and unnatural amino acids (Nϵ-2-azidoethyloxycarbonyl-L-lysine, NAEK) to evaluate its read-through efficacy. We further selected 12 lysine sites to engineer TAG or TGA PTC sites. The PTC read-through efficacy of GCE and ACE-tRNA was compared, and almost no EGFP expression was detected in the control groups. Arg-tRNA^UGA^ performed well not only at PTC-Arg sites but also at most PTC-Lys sites (Figure 3(a), upper panel). Gly-tRNA^UGA^ showed the strongest read-through ability mainly at the PTC-Gly sites (Figure 3(a), upper panel). As expected, the read-through efficacy of GCE at PTC-Lys sites was higher than that at other PTC sites, whereas the efficacy was lower than that of Arg-tRNA^UGA^ at almost all sites (Figure 3(a,b), lower panel). The MFI was evaluated using flow cytometry to quantify the read-through activities of these two systems. The results indicated that ACE-tRNAs possessed a higher PTC read-through efficacy than GCE technology, and Arg-tRNA^UGA^ performed best among all the tested ACE-tRNAs at all tested PTC sites (Figure 3(c)).

### Expanded application of GCE technology using ACE-tRNA

**Figure 4.**
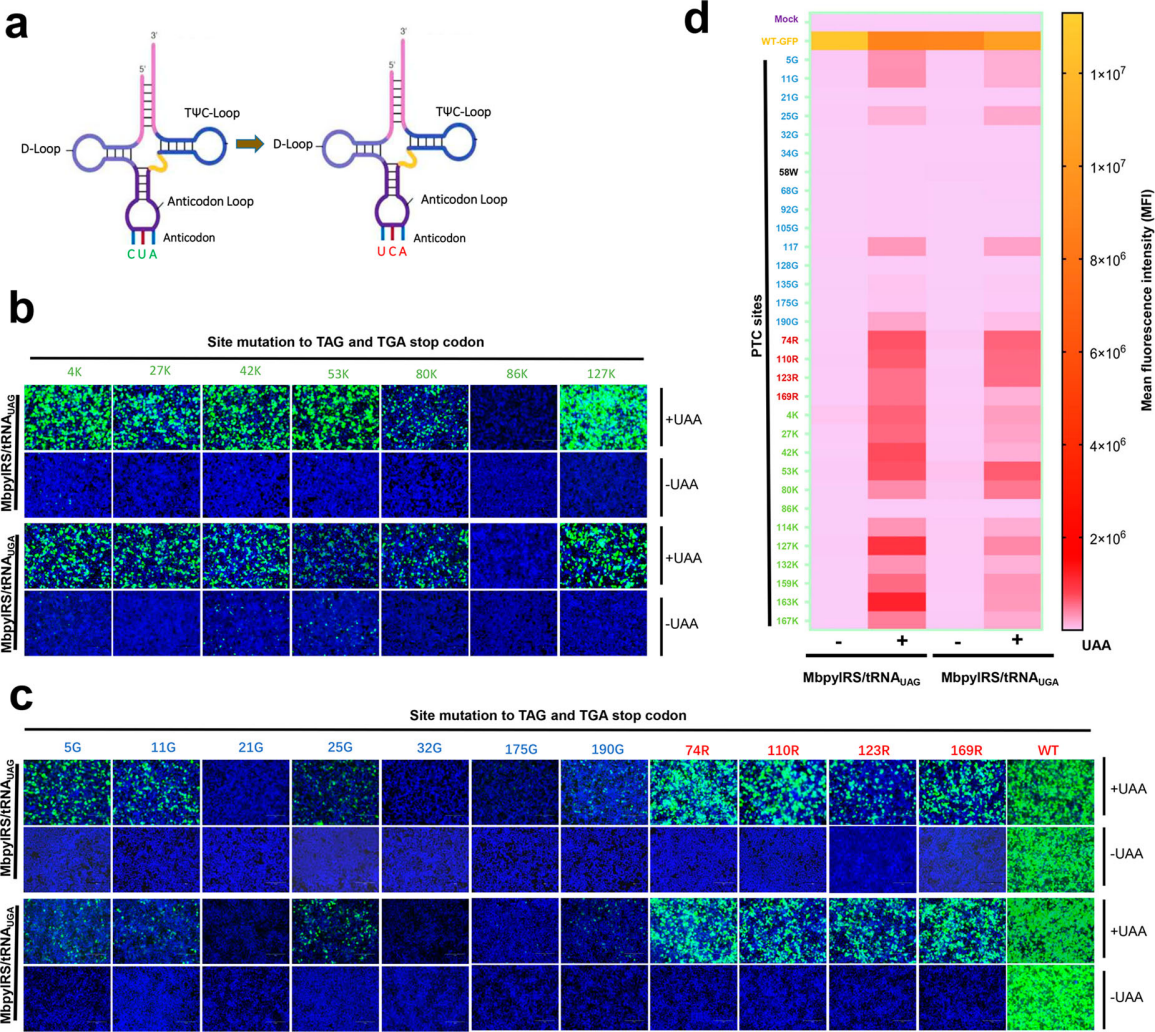
Anti-codon editing of the orthogonal tRNA used in GCE technology. (a) Schematic diagram of the anticodon editing of the orthogonal tRNA used in GCE technology. (b) and (c) The read-through efficacy of two sets of GCE systems containing tRNA_CUA_ and tRNA_UCA_ were compared. The pDC315-EGFP-TAG mutant and MbpylRS/tRNA_CUA_ pair were cotransfected into HEK-293T cells, and the pDC315-EGFP-TGA mutant and MbpylRS/tRNA_UCA_ were cotransfected into HEK-293T cells. Forty-eight hours after transfection, fluorescence microscopy was used to analyse the read-through efficacy of PTCs harbouring GFP mutants by two sets of GCE systems in the presence or absence of UAA. (d) Heatmap of the PTC read-through efficacy mediated by the two GCE systems. The MFI of EGFP was evaluated using FACS. Experiments were repeated three times, and the mean MFI is presented.

GCE always utilizes the amber codon (TAG) as the PTC site, which may limit its applied scope. Inspired by the principle of ACE-tRNA, we speculated that tRNA^Pyl^_CUA_ of GCE may be expanded by ACE-tRNA. Therefore, we generated another orthogonal translation system pair, MbpylRS-tRNA^Pyl^_UCA_, by engineering the anticodon of tRNA^Pyl^_CUA_ from CUA to UCA (Figure 4(a)). We verified whether engineered MbpylRS-tRNA^Pyl^_UCA_ pairs worked as expected by transfecting these two MbpylRS-tRNA^Pyl^ pairs together with corresponding PTC-EGFP (TAG or TGA) sites, including 12 PTC-Lys sites, 4 PTC-Arg sites, 14 PTC-Gly sites and 1 PTC-Trp site. EGFP fluorescence was only observed in the presence of NAEK (Figure 4(b,c)), and the engineered MbpylRS/tRNA^Pyl^_UCA_ showed a potent read-through ability at the corresponding PTC-EGFP sites, particularly PTC-Lys sites (Figure 4(b,c)). Finally, we also quantified the read-through activity of these PTC-EGFPs using flow cytometry, and the MFI indicated that in the presence of NAEK, both orthogonal translation systems performed well at almost all PTC-Lys and PTC-Arg sites and some PTC-Gly sites (Figure 4(d)). However, the read-through efficiency of MbpylRS-tRNA^Pyl^_UCA_ was slightly lower than that of MbpylRS/tRNA^Pyl^_CUA_ at some PTC sites. In general, our results revealed that the application scope of GCE technology was further expanded by ACE-tRNA.

### HIV-1 pseudotyped virus replication was controllable by ACE-tRNAs

**Figure 5.**
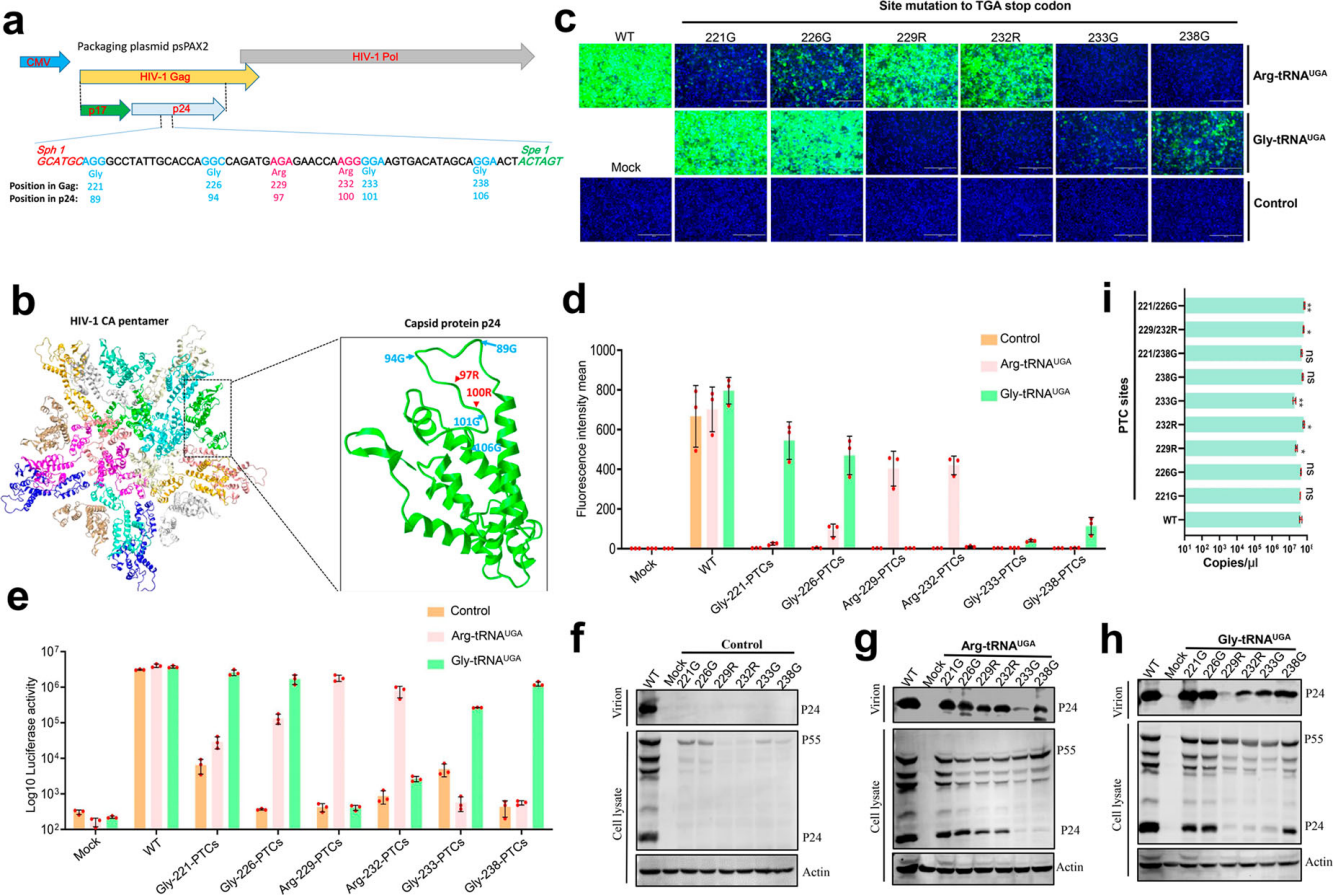
ACE-tRNAs as a precise switch for HIV-1 pseudotyped virus production. (a) Schematic diagram of the gene distribution of psPAPX2. The blue element is CMV (cytomegalovirus promoter), yellow and grey represent the HIV gag and Pol genes, respectively, and the p17 and p24 genes are indicated in the gag gene represented in green and light grey. The PTC sites were located between *SphI* and *SpeI* in p24. The numbers above the diagram represent the corresponding position in the gag protein, and the numbers below the diagram represent the corresponding position in p24. (b) Crystal structure of the HIV CA pentamer; the green part inside the box is the capsid protein p24. Amino acid codons selected for replacement with TGA are labelled within the tertiary structure of p24. (c) psPAPX2 or the indicated PTC constructs of psPAPX2, pMD2.G, PLVX-IRES-ZSGreen and the indicated ACE-tRNA or control vector were cotransfected into HEK293T cells to package the EGFP reporter virus. At 48 h posttransfection, 200 μL of collected virus were used to infect HEK293T cells. Fluorescence was observed at 48 h postinfection (hpi). An untransfected group was used as a mock control. Cell nuclei were stained with DAPI; scale bar 200 μm. (d) The MFI of infected HEK293T cells was evaluated using flow cytometry. (e) Firefly luciferase assay of ACE-tRNAs in the production of luciferase reporter PTC-containing virus. Two hundred microliters of collected virus were used to infect HEK293T cells, and the cells were washed and lysed for luciferase activity detection at 48 hpi. Experiments were repeated at least three times. Error bars represent the standard deviations (SD). (f) Western blot detection of PTC-harbouring virus production with the control vector, (g) Arg-tRNA^UGA^ or (h) Gly-tRNA^UGA^. Experiments were repeated at least three times, and a representative result is shown.

HIV-1 was used as a model RNA virus to determine whether ACE-tRNAs can serve as a precise switch for virus replication. For safety concerns, we used pseudotyped HIV-1 in the current study and modified psPAX2 to facilitate PTC engineering, which did not alter the efficacy of HIV-1 pseudotyped virus packaging (Figures S2a, S2b and S2c). We chose four glycines at positions 221, 226, 233, and 238, as well as two arginines at positions 229 and 232, of the gag protein for PTC engineering (Figure 5(a)). The HIV-1 gag polyprotein (p55) is processed into p24 and p17 during HIV-1 replication, and the positions of PTC-modified sites are labelled (Figure 5(a)). The structure of the HIV-1 capsid pentamer in intact virus is shown, and all potential PTC engineering sites were labelled (Figure 5(b)) [28]. Pseudotyped HIV-1 was generated by cotransfection with three plasmids, the transfer plasmid PLVX-IRES-ZSGreen harbouring the EGFP reporter gene pM2.G (a VSV-G coding plasmid) and psPAX2 into HEK293T cells. Therefore, HIV-1 containing the PTC was rescued by cotransfecting these plasmids together with the indicated ACE-tRNA plasmid. The packaging virus in the supernatant was collected at 48 h posttransfection, and WT HIV-1 pseudotyped virus (as a positive control) and the indicated PTC-containing virus were used to infect HEK293T cells and assess the ACE-tRNA read-through activity. We found that Arg-tRNA^UGA^ and Gly-tRNA^UGA^ successfully controlled HIV-1 pseudotyped PTC-containing virus production. Arg-tRNA^UGA^ exhibited potent PTC read-through activity and successfully produced an infectious PTC-Arg-229 virus and PTC-Arg-232 virus (Figure 5(c)). Furthermore, Gly-tRNA^UGA^ performed well in PTC-Gly-221 and PTC-Gly-226; however, viral production was lower for PTC-Gly-233 and PTC-Gly-238 (Figure 5(c)). We next used flow cytometry to quantify PTC-containing virus production by evaluating the MFI in virus-infected HEK293T cells (Figure 5(d)). Because firefly luciferase is more sensitive than EGFP, we further confirmed the production of the PTC-containing virus by measuring the activity of the firefly luciferase reporter, and a similar result was obtained (Figure 5(e)). Based on these results, ACE-tRNA successfully restores the production of HIV-1 PTCs, similar to the WT virus. Finally, the production of HIV-1 PTCs at the protein level was determined, and we detected very little p24 in the cell lysate or virions in the supernatant of the control vector group (Figure 5(f)). However, extremely low levels of p55 were detectable in cell lysates (Figure 5(f)). In contrast, in the presence of Arg-tRNA^UGA^ and Gly-tRNA^UGA^, p55 expression in PTC viruses in cell lysates was noticeably increased compared to that in the control group (Figure 5(g,h)). For the Arg-tRNA^UGA^ group, PTC-containing virions were successfully produced in the supernatant of the PTC-Arg-229 and PTC-Arg-232 viruses (Figure 5(g)). In addition, Arg-tRNA^UGA^ restored the production of PTC-containing virions by all four Gly-PTC viruses to varying degrees (Figure 5(g)). Next, we further evaluated the effect of Gly-tRNA^UGA^ on PTC-containing virion production, and the results indicated that it performed well in the production of Gly-221-PTC virus, Gly-226-PTC virus, Gly-233-PTC virus and Gly-238-PTC virus. Moreover, Gly-tRNA^UGA^ rescued both Arg-229-PTC and Arg-232-PTC viruses at a low level (Figure 5(h)). Furthermore, we also detected the mRNA levels of these PTC-containing viruses and found that NMD and the PTC site preference collaborate to determine the PTC read-through activity of ACE-tRNAs (Figure 5(i)). We attempted to introduce two PTC sites in HIV-1 gag to improve the safety of the PTC-containing virus, and the results indicated that the double Arg-PTC virus was successfully rescued in the presence of Arg-tRNA^UGA^ or Gly-tRNA^UGA^ (Figures S3a, S3b, S3c, S3d, S3e). However, the efficiency of read-through activity was slightly affected compared to the virus containing a single PTC site. Taken together, ACE-tRNAs can serve as a precise switch for pseudotyped HIV-1 production.

### Design and generation of PTC-PRV

**Figure 6.**
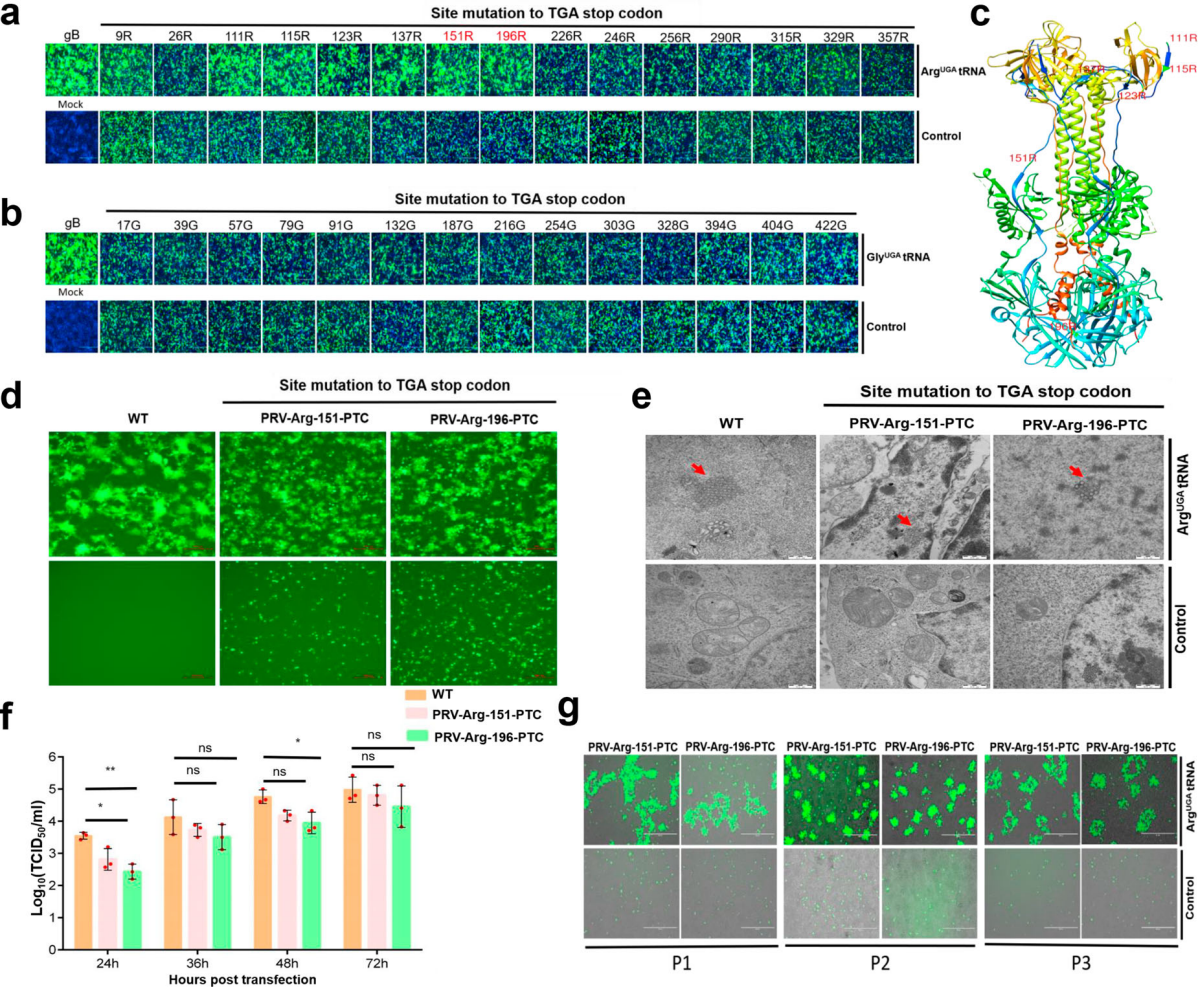
ACE-tRNAs as a precise switch for PRV-PTC replication. (a) A cell-to-cell fusion assay was used to evaluate the read-through efficiency of PTC-harbouring gB mutants by Arg-tRNA^UGA^ or (b) Gly-tRNA^UGA^. A total of 150 ng (each) of plasmids encoding gB or its mutants, gD, gH, gL, pDC315-EGFP and 400 ng of the indicated ACE-tRNA were cotransfected into RK13 cells growing in 24-well plates. In parallel, transfection of the ACE-tRNA empty vector pUC57 with these plasmids was performed in each group as a control. Fluorescence microscopy was used to analyse the read-through efficacy with the indicated gB-mediated cell fusion analysis 48 h after transfection. The wild-type gB-transfected group served as the positive control, and the nontransfected group served as the mock control. Nuclei were stained with DAPI, scale bar = 200 μm. (c) The corresponding position of PTC sites with good fusion and expression effects in the gB crystal structure. (d) Rescue of PRV-PTC virus in HEK-293T cells. pPRV-Arg-151-PTC and pPRV-Arg-196-PTC were cotransfected with 2 µg of Arg-tRNA^UGA^ or pUC57 plasmid into cells. The 2 µg pPRV-Bac transfection group was used as a positive control, and the untransfected group was used as a mock control. At 48 hpi, the CPE phenotype was observed using fluorescence microscopy. (e) Electron microscopy was used to observe the morphology of PRV-PTC and PRV-WT. Scale bar = 50 nm. (f) The TCID_50_ was calculated to determine the titre of PRV-PTC rescued at different time points. Experiments were repeated at least three times. Error bars represent the SD. (g) Verification of the genetic stability of progeny PTC-harbouring viruses by passaging in HEK-293T cells expressing Arg-tRNA^UGA^ or the control cells.

Next, we used PRV as a model DNA virus and attempted to generate PTC-harbouring PRV. *gB* is an essential gene for PRV replication, and it was selected to engineer potential PTC sites and evaluate the PTC read-through efficacy of Arg-tRNA^UGA^ and Gly-tRNA^UGA^[17]. In the current study, the PTC of the opal codon was introduced in the *gB* coding region, including 15 arginine sites and 14 glycine sites. *gB*, *gD*, *gH* and *gL* are essential viral genes for virus-mediated cell-to-cell fusion, which is a key step for PRV spread. A cell-to-cell fusion assay was applied to test whether these PTC sites were efficiently read through by ACE-tRNA. A transient transfection-based cell-to-cell fusion assay was performed, and the results indicated that among the 15 PTC-Arg sites, 111R, 115R, 123R, 137R, 151R and 196R successfully and efficiently induced syncytium formation in the presence of Arg-tRNA^UGA^, and no syncytia formed without Arg-tRNA^UGA^ (Figure 6(a)). The corresponding PTC positions in the gB crystal structure are shown (Figure 6(c)). Interestingly, we did not observe any syncytia phenotype in the presence of Gly-tRNA^UGA^ at any of the attempted PTC-Gly sites (Figure 6(b)). Therefore, Arg-tRNA^UGA^ can be used as a precise switch for controlling PRV gB protein expression. Then, we performed Western blotting to further confirm the read-through activity (Figures S4a to S4h). The results were consistent with the cell-to-cell fusion assay. Overall, Arg-tRNA^UGA^ is an ideal switch for controlling gB protein expression.

Next, we selected 151R and 196R as potential PTC engineering sites and subsequently generated PTC-harbouring infectious clones, pPRV-Arg-151-PTC and pPRV-Arg-196-PTC. We rescued the PTC-harbouring virus by cotransfecting HEK-293T cells with pPRV-Arg-151-PTC or pPRV-Arg-196-PTC with or without Arg-tRNA^UGA^. Forty-eight hours later, typical PRV-induced syncytia formed in the presence of Arg-tRNA^UGA^, and no syncytia were detected in the control group (Figure 6(d)). We also successfully rescued the PTC-containing virus in Vero cells (Figures S5a and S5b). Electron microscopy analysis was performed to further confirm that PRV-PTC was rescued, as expected, and we observed PRV particles in the presence of Arg-tRNA^UGA^ (Figure 6(e)); however, no PRV particles were observed in the control group (Figure 6(e)).

Next, we evaluated the growth kinetics of PRV-PTC, and the viral titre of PRV-PTC was similar to that of the parental PRV-WT strain (Figure 6(f) and Figures S6a, S6b). In addition, we evaluated the genetic stability of the PTC-harbouring virus by serially passaging PRV-PTC 3 times, and a typical PRV-induced CPE only occurred in the presence of Arg-tRNA^UGA^ but not in the control group (Figure 6(g)). Furthermore, the *gB* sequence of the PTC-PRV of each passage was also verified by DNA sequencing, and all the PRV-PTCs maintained their respective PTCs in their original position. These results indicated that PRV-PTC was genetically stable *in vitro* (Figures S7a, S7b and S7c).

### Evaluation of the safety of PRV-PTC *in vivo*

**Figure 7.**
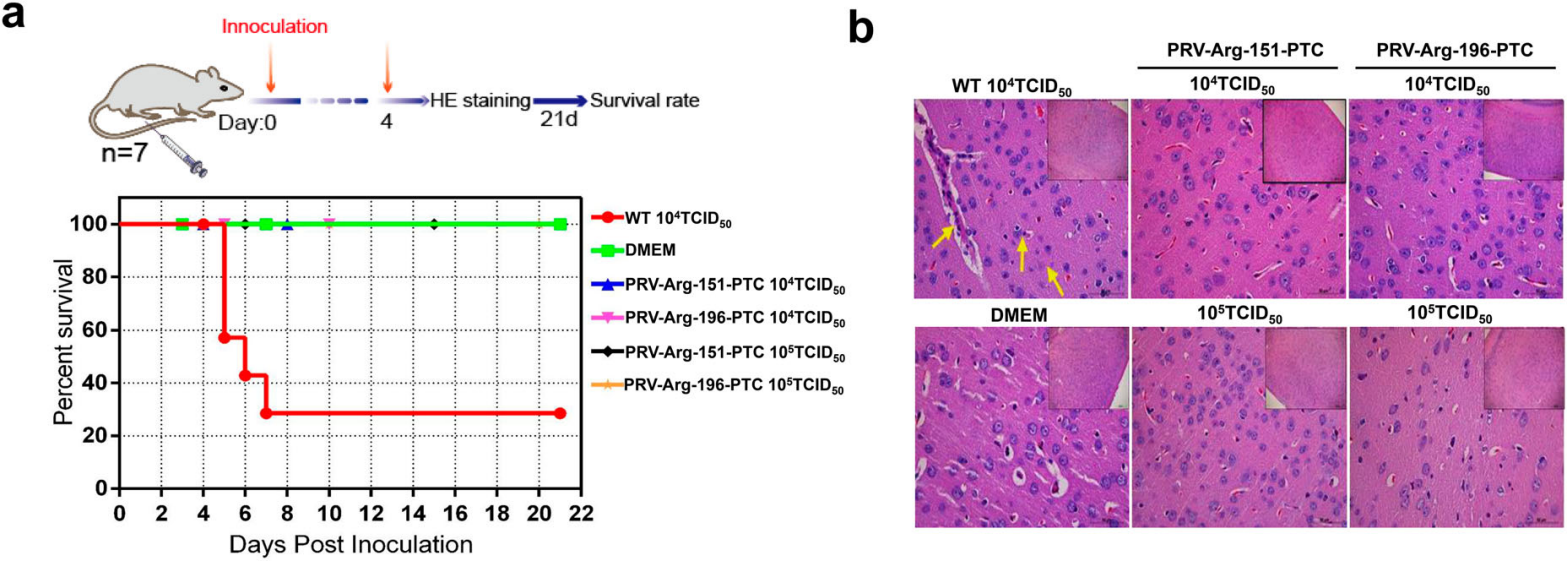
Characterization of the safety of PRV-PTC *in vivo*. (a) Percent survival of mice inoculated with different agents (*n* = 7) over 21 days. (b) H&E staining of the brains of the mice in different groups. Yellow arrows indicate representative abnormal areas, scale bars, 50 μm (magnified images of H&E staining), and the top right insets show full images, scale bars, 100 μm.

The safety of PRV-PTC was evaluated *in vivo*. BALB/c mice were infected with PRV-Arg-151-PTC or PRV-Arg-196-PTC; furthermore, the wild-type PRV and DMEM groups were used as positive or negative controls. A total of 71.4% of mice (five of seven mice) died when infected with 1 × 10^4^ TCID_50_ wild-type PRV; in contrast, no mice died in the PRV-Arg-151-PTC and PRV-Arg-196-PTC virus groups infected with a dose of 1 × 10^4^ TCID_50_ or even at a higher dose of 1 × 10^5^ TCID_50_ (Figure 7(a)). No typical PRV infection symptoms were observed (data not shown). The mouse brains were collected for the histopathological examination, and the results confirmed that the brain manifested perivascular lymphocyte infiltration, nerve cell necrosis and mild neutrophilia in the wild-type PRV group (Figure 7(b)). However, no obvious brain lesions were observed in the PRV-PTC infection and control groups.

### Evaluation of the immunogenicity of PRV-PTC *in vivo*

**Figure 8.**
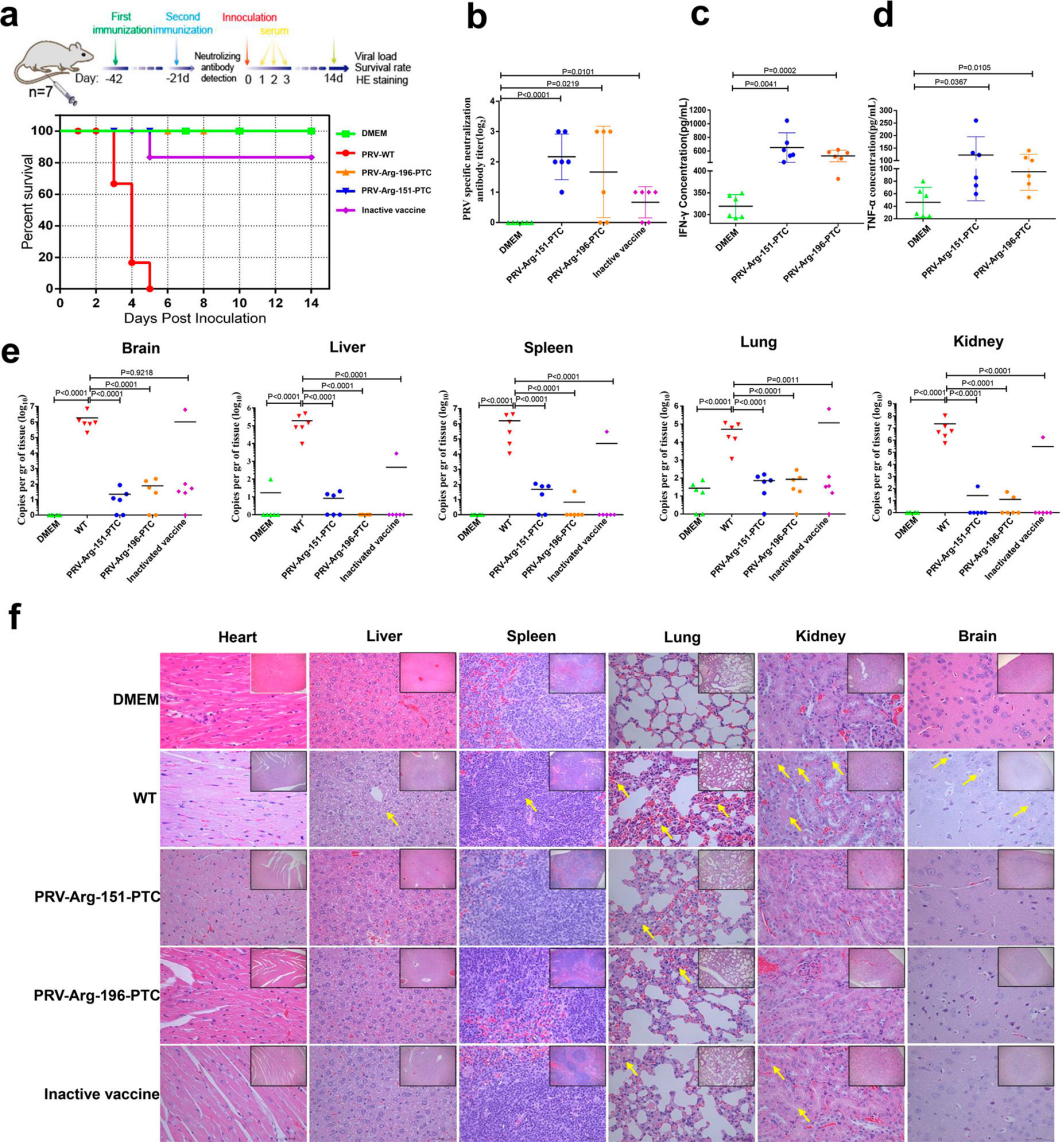
Characterization of the immunogenicity of PRV-PTC *in vivo*. (a) Percent survival of immunized mice after challenge with a lethal dose of the PRV strain JS2012 (*n* = 6). (b) PRV-specific neutralizing antibody titres in serum. Sera were collected from two immunized BALB/c mice 1 d before challenge with the PRV JS2012 strain (*n* = 6). Bars indicate SD. (c) Serum IFN-γ and (d) TNF-α levels. Serum was obtained from mice immunized two times with the indicated viruses 1 d after the challenge infection (*n* = 6). The serum was tested for the levels of IFN-γ and TNF-α using ELISA. The values are presented in pg IFN-γ per mL, and the bars indicate standard deviations. (e) Viral loads in the heart, liver, spleen, lung, kidney and brain of each group (*n* = 6). The mice were dissected at 14 dpi to collect these tissues. After homogenization, the tissue samples were used for DNA extraction and RT–qPCR. The values are presented in virus copies per gram, and the mean values are indicated. (f) H&E staining of hearts, livers, spleens, lungs, kidneys and brains of the mice in different groups after dissection at 14 dpi. Yellow arrows indicate representative abnormal areas. Scale bars, 50 μm (magnified images of H&E staining), and the top right insets show full images, scale bars, 100 μm.

Next, we evaluated the possibility of using PRV-PTC as a potential vaccine candidate. The mice were immunized twice with 1 × 10^5^ TCID_50_ of PRV-PTC or inactivated PRV. Three weeks after the second immunization, the mice were challenged with 3 × 10^4^ TCID_50_ of wild-type PRV. Mice in the control group died after wild-type PRV infection, and all mice exhibited severe neurological symptoms (Figure 8(a)). In the inactivated PRV-immunized group, 1/5 mice died after wild-type PRV challenge. However, in the PRV-PTC-immunized group, no mice (0/5) died or displayed typical symptoms. Based on these results, PRV-PTC provided full protection against wild-type PRV challenge, and the protection rate was higher than that of inactivated PRV (Figure 8(a)). Furthermore, the titre of the specific neutralizing antibody against PRV was evaluated after the second immunization, and we found that the PRV-PTC-immunized group produced higher levels of PRV-specific neutralizing antibodies than the inactivated PRV group (Figure 8(b)). The production of IFN-γ (Figure 8(c)) and TNF-α (Figure 8(d)) in serum was detected using ELISA and was significantly improved compared to that in the control. We also evaluated the viral load in the indicated organs. In the control group immunized with DMEM, the viral load was very high, while the viral loads were extremely low in the PRV-Arg-151-PTC- and PRV-Arg-196-PTC-immunized groups (Figure 8(e)). The viral load of the inactivated PRV-immunized group was also good, except in the mice that died.

We also evaluated histopathological changes by H&E staining. Our results showed that the dead mice in the DMEM group developed severe microscopic lesions in multiple organs, such as (1) severe and extensive mild inflammatory cell infiltration in the lungs, (2) extensive necrosis in the brain, (3) widely denatured hepatocytes around the central vein in the liver, and (4) white pulp hyperplasia, partial lymphocyte cluster necrosis in the spleen and renal tubular epithelial cell detachment with a small amount of pipe type in the kidney (Figure 8(f)). In contrast, the inactivated PRV and PRV-PTC immunized groups exhibited fewer histopathological changes in the lungs and no apparent histopathological changes in other organs (Figure 8(f)). Thus, in general, both PRV-PTC-immunized groups exhibited better conditions than the inactivated PRV-immunized group.

## Discussion

Despite notable successes of LAVs and inactivated viruses, the safety of LAVs and immunogenicity of inactivated viruses always raise concerns. Here, we provide proof-of-principle evidence that ACE-tRNAs rapidly attenuated pathogenic viruses, and this strategy takes into account safety and efficacy. In previous studies, PTC-harbouring viruses were produced using GCE technology [12-16]. GCE technology depends on the cooperation of the orthogonal translation machinery of the tRNA synthetase/tRNA^CUA^ pair and UAA. Indeed, PTC-containing viruses generated using GCE were replication-incompetent in conventional cells, whereas viruses attenuated using other strategies still replicated. The UAA incorporation efficacy seems extremely low for some viruses [16,17]. The current study is the first to apply genetically modified tRNA to generate PTC-harbouring viruses. This method is simpler than GCE technology, as it only requires a genetically modified tRNA. Another advantage of the ACE-tRNA method is that the engineered tRNA produces a PTC-harbouring virus with a cognate amino acid that generates a natural viral antigen. However, the antigen produced by the GCE system carries unnatural amino acids, which may affect its antigenicity. In addition, ACE-tRNA exhibits minimal read-through activity at natural protein termination codons, which will have no effect on the physiological activity of cells [18]. Overall, we propose that genetically modified tRNA technology has more advantages than GCE technology in generating PTC-harbouring viruses in the future.

The incorporation of unnatural amino acids into amber stop codons by GCE is not as efficient as that of sense codons [29]. Here, we showed that the ACE-tRNA was more efficient than GCE in generating the PTC-containing virus; moreover, its efficiency might be further improved, such as increasing the copy number of ACE-tRNA genes and using robust promoters that increase the expression of ACE-tRNA [30,31]. Furthermore, PTC read-through is limited by release factors that mediate peptide chain termination, and evolved ribosomes may improve ACE-tRNA read-through efficiency [32]. Knockdown or an engineered release factor may also increase the read-through activity of ACE-tRNA [33,34].

The PTC read-through activity of three ACE-tRNAs, Arg-tRNA^UGA^, Gly-tRNA^UGA^ and Trp-tRNA^UGA^, was evaluated in the current study. More engineered tRNA should be screened and evaluated in the future to select the most effective ACE-tRNA. Furthermore, as ACE-tRNAs have an obvious PTC site preference, systematic selection of potential sites will increase the yields of PTC-harbouring viruses. Concerning the safety of PTC-containing viruses, one PTC site may be mutated by the potential reversion of the opal codon to a sense codon during PTC-containing virus replication; therefore, two PTC sites can be applied in PTC-containing viruses to increase their safety. The effect of PTC site combinations on the high efficiency of ACE-tRNA read-through and PTC-harbouring virus production requires further study.

A vaccine used as a biological product to protect the host against infection and/or disease must safely induce an immune response [35]. The application of ACE-tRNA-generated PTC-harbouring viruses showed promising results in terms of both safety and immunogenicity [12]. As a replication-incompetent virus, the replication of the PTC-containing virus relies on the presence of ACE-tRNA, which is an artificial tRNA that does not exist in mammalian cells. This unique ACE-tRNA-dependent feature allows the PTC-harbouring virus to safely function as a nonreplicating vaccine that does not induce a mild disease, similar to some live attenuated vaccines [36]. We found that both PRV-PTC were nonvirulent in mice, while infection with the parental PRV killed mice rapidly (Figure 7(a)). Compared to the parental virus, the engineered PTC-containing virus showed a similar infectious capacity and infected and activated the host's immune response without viral replication. The advantage of the PTC-harbouring virus to viral vector vaccines and nucleic acid vaccines is that it contains all natural viral antigens, similar to wild-type viruses, needed to elicit a host immune response [12]. Compared with mRNA vaccines and subunit recombinant protein vaccines, the protective antigen must be identified first. However, for some viruses, especially emerging viruses, we do not know about which gene(s) was/were protective antigen(s). The advantage of this technology is that we do not need to know this information. Furthermore, subunit recombinant protein vaccines may induce a strong antibody response and a limited T cell response. The PTC-containing virus can enter cells and theoretically induce a potent T cell response. Our data confirmed that PRV-PTC elicited a robust immune response and provided complete protection against wild-type PRV challenge (Figure 8(a)). Therefore, the PTC-harbouring virus generated using modified tRNA technology can be applied as a vaccine candidate with excellent safety and immunogenicity.

The current study also has some limitations, and we hope these limitations will be overcome in future studies. First, if the PTC-containing virus is used for vaccine manufacturing, an engineered cell line that stably expresses the ACE-tRNA must be constructed to ensure mass production of the PTC-harbouring virus, and the stability of the PTC-containing virus during passage should be further verified. Second, the durability of neutralizing antibody and PRV-specific T cell responses was not evaluated. Serum IFN-γ and TNF-α levels detected in this study are not indicators of specific cellular immune responses. Third, we do not know what factors determine the different read-through capacities of different PTC sites. Based on the results from the current experiments, we recognized that this property is related to PTC-engineered proteins, the ACE-tRNA used and NMD. We hope to determine some rules and corresponding possible mechanisms in the future.

Overall, the notable challenge of vaccine development to overcome in the future is the need to swiftly respond to emerging pathogen outbreak situations and diseases with high genetic diversity [35]. Therefore, the development of technologies that are flexible and facilitate the rapid development and production of vaccines is urgently needed [37]. In this study, we developed a new engineered transfer RNA-based vaccine platform to generate PTC-harbouring viruses. This platform will facilitate rapid vaccine development, which may contribute to fighting emerging or re-emerging viral threats in the future.

## Supplementary Material

Supplemental Material
