# Supplementary material for: A novel viral vaccine platform based on engineered transfer RNA
Source: Emerg Microbes Infect. 2022 Dec 18;12(1):2157339. doi: 10.1080/22221751.2022.2157339 (PMC9769134; doi:10.1080/22221751.2022.2157339)
Supplement: Supplemental Material [file TEMI_A_2157339_SM5875.docx]

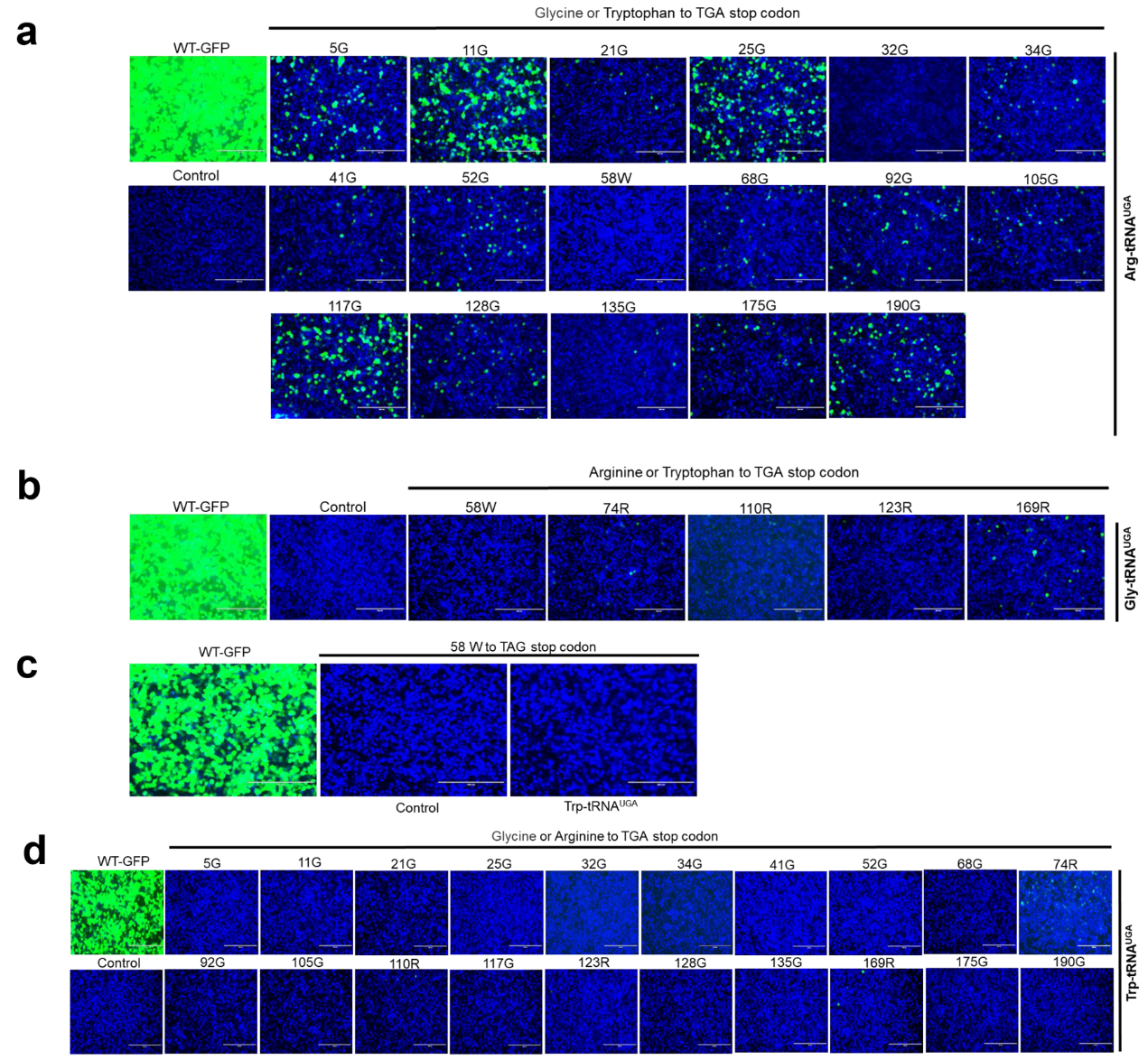


**Figure S1. ACE-tRNA read-through activity of PTC-EGFP.** **(a) Gly/Trp-PTC read-through by Arg-tRNA^UGA^.** HEK293T cells were co-transfected with 1 μg Arg-tRNAUGA and 1 μg Gly or Trp PTC of EGFP. At 48 h post-transfection, fluorescence microscopy was used to evaluate the ACE-tRNA suppression efficacy. **(b)** Arg/Trp-PTC read-through by Gly-tRNA^UGA^. HEK293T cells were co-transfected with 1 μg Gly-tRNA^UGA^ and 1 μg Arg or Trp-PTC of EGFP. At 48 h post-transfection, fluorescence microscopy was used to evaluate the ACE-tRNA suppression efficacy. **(c)** Trp-PTC read-through by Trp-tRNA^UGA^. HEK293T cells were co-transfected with 1 μg Trp-tRNA^UGA^ and 1 μg Trp-PTC EGFP. At 48 h post-transfection, fluorescence microscopy was used to evaluate the ACE-tRNA suppression efficacy. **(d)** Arg/Gly-PTC read-through by Trp-tRNA^UGA^. HEK293T cells were co-transfected with 1 μg Trp-tRNA^UGA^ and 1 μg PTC EGFP. At 48 h post-transfection, fluorescence microscopy was used to evaluate the ACE-tRNA suppression efficacy. The wild-type EGFP group was used as a positive control, the non-transfected group was used as a mock control, and the pDC315-EGFP PTC plasmid and ACE-tRNA empty vector pUC57 co-transfection groups were used as negative controls. Cell nuclei were stained with DAPI; scale bar=200 μm.


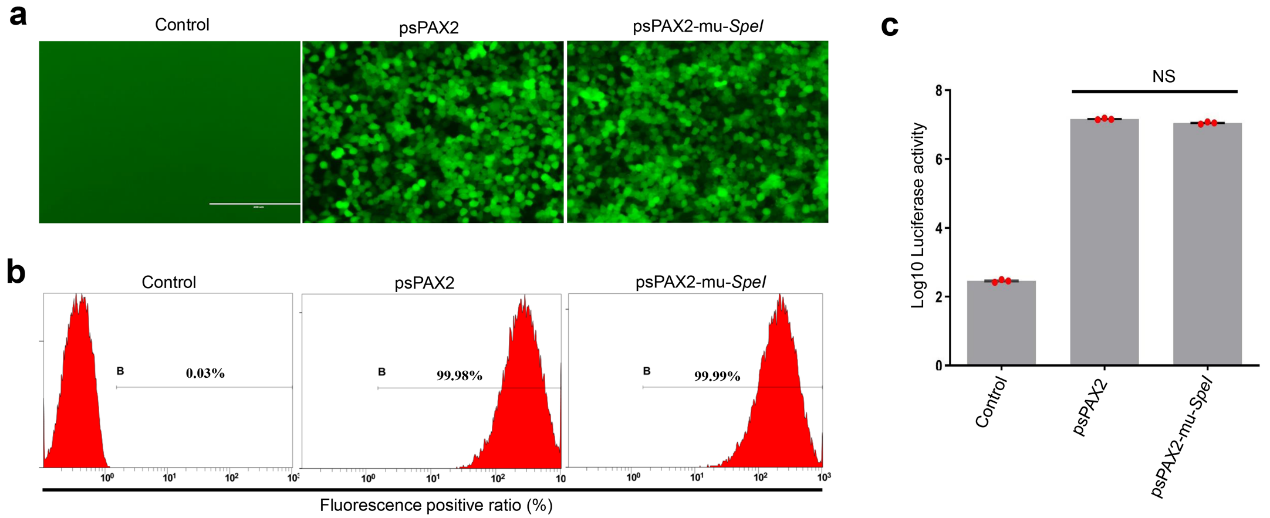


**Figure S2. Modification of the *Spe I* restriction site has no effect on the production of pseudotyped HIV-1.** **(a)** An EGFP reporter of pseudotyped HIV-1 was generated in HEK293T cells by cotransfecting WT psPAPX2 or psPAPX2-mu-SpeI with PMD2.G and PLVX-IRES-ZSGreen; viruses were collected 48 h post-transfection. 200 µL of collected virus was used to infect HEK293T cells in 24-well plates per well. Fluorescence microscopy was used to evaluate the production of pseudotyped virus. Scale bar was 200 μm. **(b)** Infectivity was analyzed by flow cytometry. **(c)** A luciferase reporter of pseudotyped HIV-1 was generated in HEK293T cells by co-transfecting WT psPAPX2 or psPAPX2-mu-SpeI with PMD2. G and HIV-l-Luc, viruses were collected 48 h post-transfection. Two hundred microliters of collected virus was used to infect HEK293T cells in 24-well plates per well; after 48 h, the cells were washed and lysed for luciferase activity detection. Experiments were repeated at least three times. Error bars represent the standard error (SD); NS represents no significant difference.


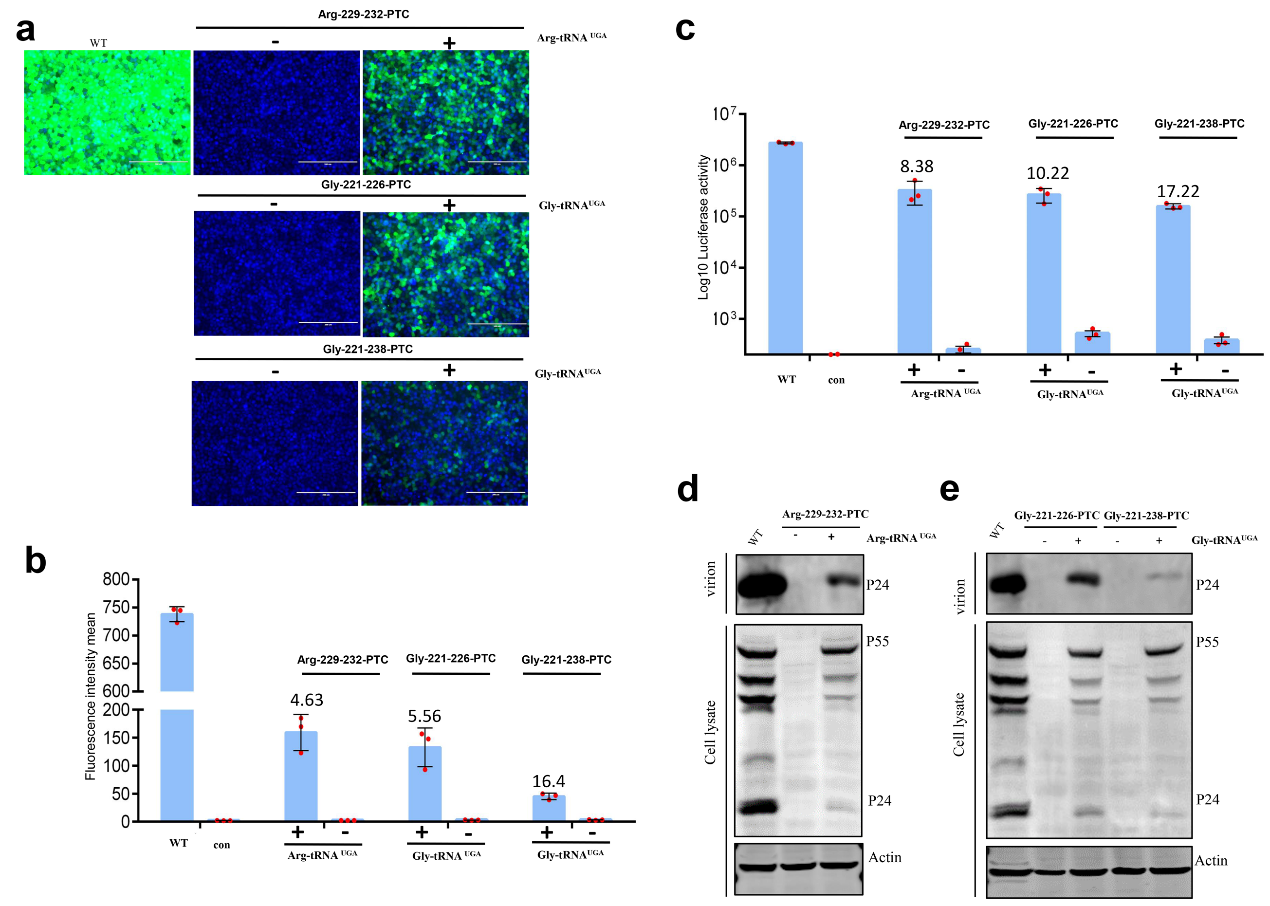


**Figure S3. Arg-tRNA^UGA^ and Gly-tRNA^UGA^ rescue PTC viruses with double Arg-PTC sites.** (**a**) psPAPX2 or the indicated PTC constructs of psPAPX2, pMD2.G, PLVX-IRES-ZSGreen and the indicated ACE-tRNA^UGA^ or control vector were co-transfected into HEK293T cells to package pseudotyped HIV-1 EGFP reporter virus. At 48 h post-transfection, 200 μL of collected virus was used to infect HEK293T cells. Fluorescence was observed at 48 h post infection (hpi). Cell nuclei were stained with DAPI; scale bar 200 μm. (**b**) The mean fluorescence intensity of infected HEK293T cells was evaluated by flow cytometry. (**c**) Firefly luciferase assay for Arg-tRNA^UGA^ and Gly-tRNA^UGA^ in the production of luciferase reporter PTC virus. 200 μL of collected virus was used to infect HEK293T cells, and the cells were washed and lysed for luciferase activity detection at 48 hpi. Experiments were repeated at least three times. Error bars represent the standard error (SD). (**d**) Western blot detection of PTC virus production of the control vector and Arg-tRNA^UGA^. (**e**) Western blot detection of PTC virus production of the control vector and Gly-tRNA^UGA^ . Experiments were repeated at least three times, and a representative result is shown.


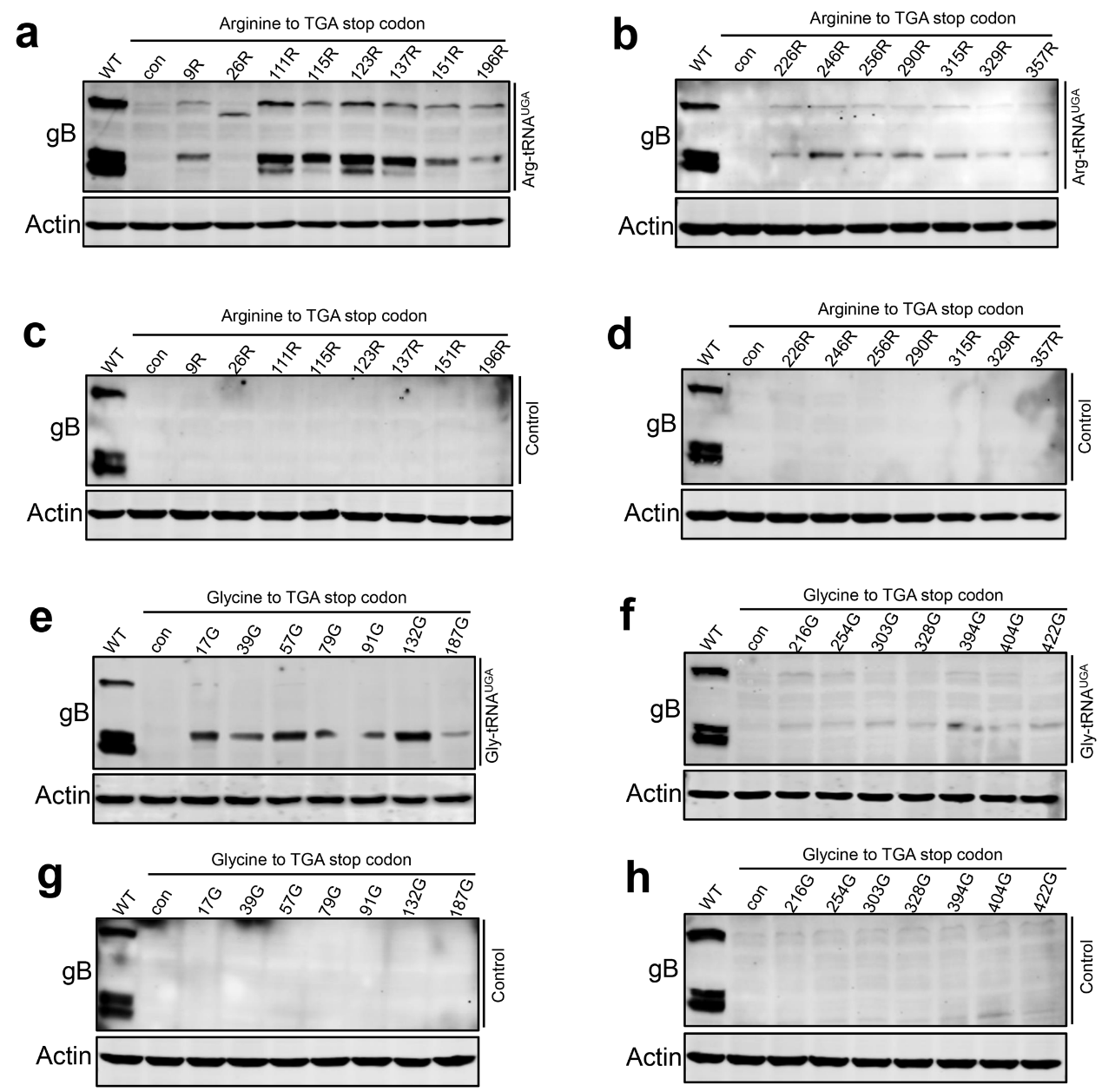


**Figure S4. Read-through efficacy for gB PTC by ACE-tRNA identified by Western blot.** HEK293T cells in good growth condition were plated in 24-well plate (4x10^5^cells/well). 0.75 μg pCAGGS-gB with arginine replaced by PTC were co-transfected with 0.75 µg **(a, b)** Arg-tRNA^UGA^. **(c, d)** Empty vector pUC57. 0.75 μg pCAGGS-gB with glycine replaced by PTC were co-transfected with 0.75 µg **(e, f)** Gly-tRNA^UGA^. **(g, h)** Empty vector pUC57. 0.75 μg pCAGGS-gB and 0.75 µg pUC57 transfection group were used as positive control, and the non-transfection group was used as mock control.


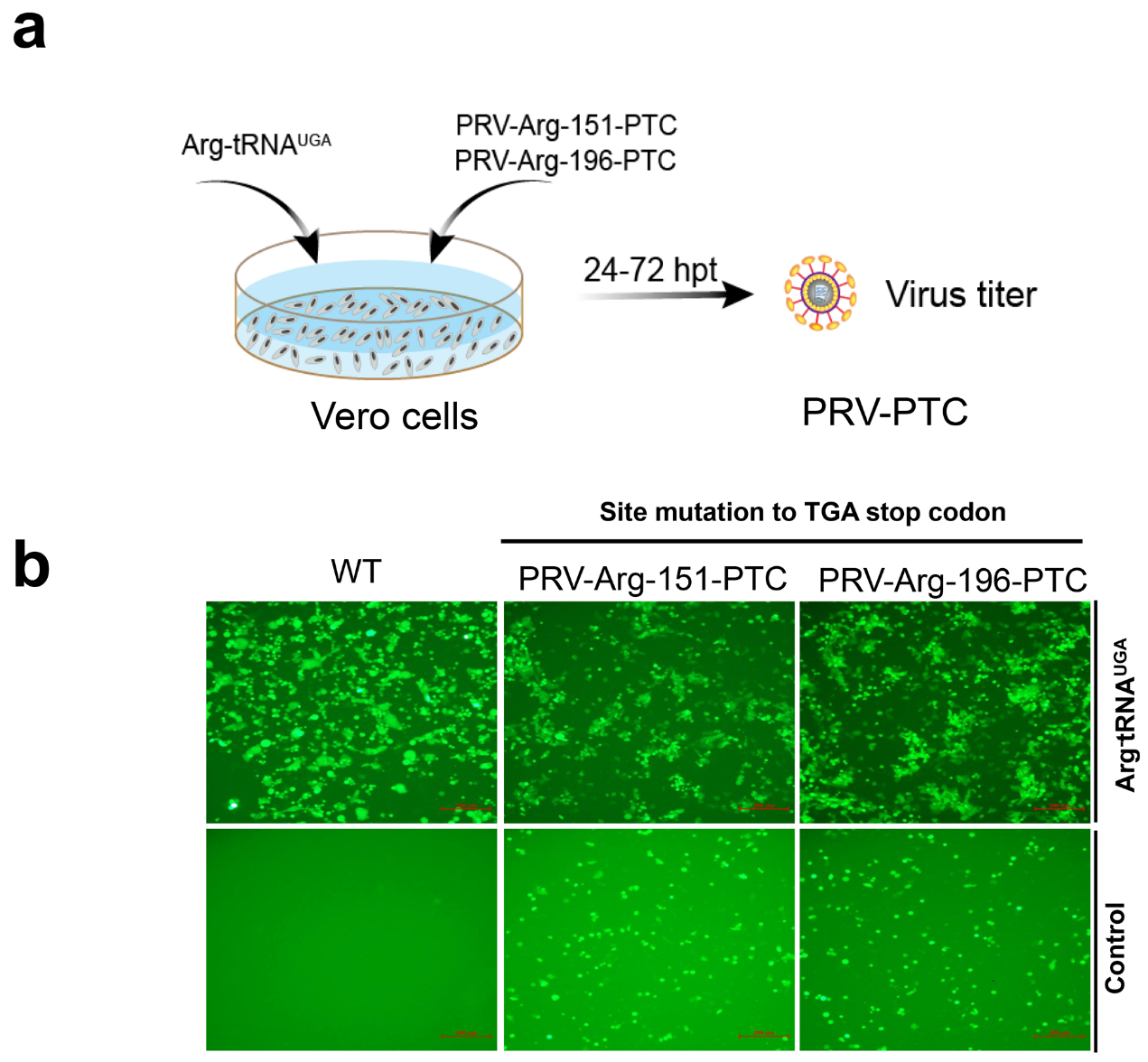


**Figure S5. Rescue of PRV-PTC virus in Vero cells.** **(a)** Schematic illustration of rescue of PRV-PTC virus in Vero cells. **(b)** 2 µg pPRV-Arg-151-PTC, pPRV-Arg-196-PTC was co-transfected with 2 µg Arg-tRNA^UGA^ or pUC57 plasmid into cells respectively. 2 µg pPRV-Bac transfection group was used as positive control, the untransfected group was used as mock. 48 hpi, the CPE phenotype was observed by fluorescence microscopy, scale bar = 200 μm.


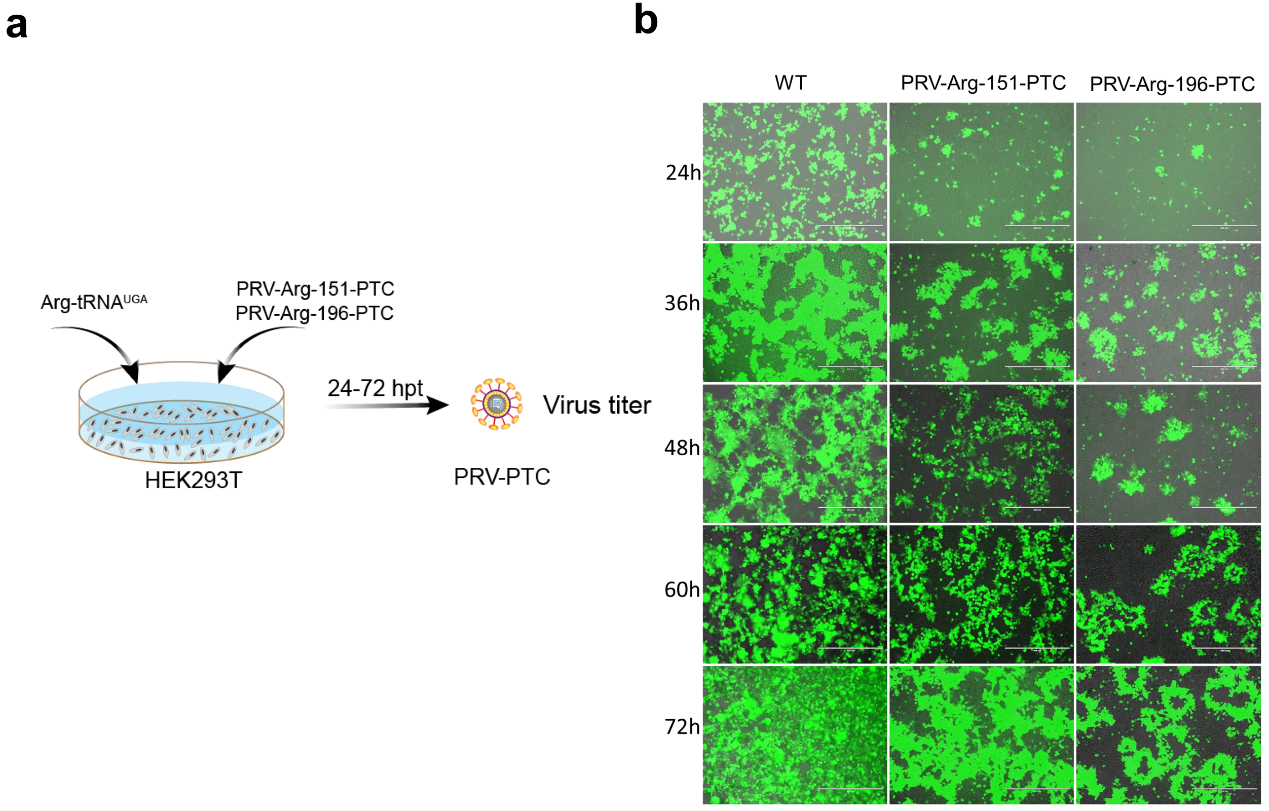


**Figure S6. Fluorescence microscopy was used to observe the replication of PRV-PTC virus at different time points. (a)** Schematic illustration of rescue of pPRV-PTC transfection in HEK293T cells. **(b)** pPRV-Arg-151-PTC or pPRV-Arg-196-PTC was co-transfected with 2 µg Arg-tRNA^UGA^ into HEK293T cells respectively. 2 µg pPRV-Bac transfection group was used as positive control. The CPE phenotype was observed by fluorescence microscopy at 24 h, 36 h, 48 h, 60 h, 72 h, scale bar = 400 μm.


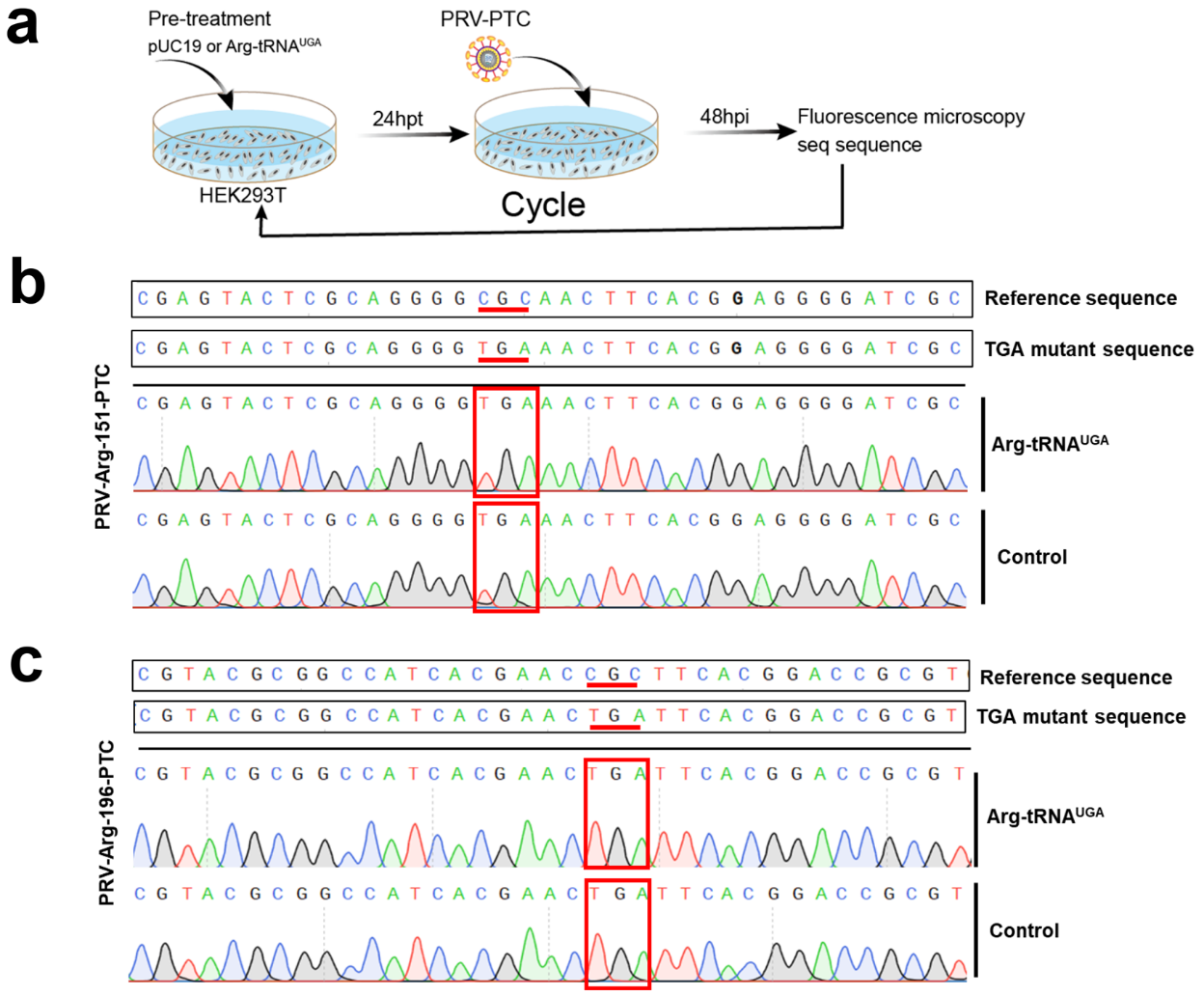


**Figure S7. Verification of genetic stability by sequencing.** **(a)** Schematic illustration of PRV-PTC virus passaging in HEK293T cells. The third passage sequence of **(b)** PRV-Arg-151-PTC and **(c)** PRV-Arg-196-PTC virus in the presence of Arg-tRNA^UGA^ or not.

**Table S1. Primers for construction of TGA harboring pDC-315-GFP mutants**

| Primer | Primer Sequence 5’→3’ | |
| --- | --- | --- |
| 5G-TGA-F | | ATGGTGAGCAAGTGAGAGGAGCTGTTCACC |
| 5G-TGA-R | | GGTGAACAGCTCCTCTCACTTGCTCACCAT |
| 11G-TGA-F | | GAGGAGCTGTTCACCTGAGTGGTGCCCATCCTG |
| 11G-TGA-R | | CAGGATGGGCACCACTCAGGTGAACAGCTCCTC |
| 21G-TGA-F | | CTGGTCGAGCTGGACTGAGACGTAAACGGCCAC |
| 21G-TGA-R | | GTGGCCGTTTACGTCTCAGTCCAGCTCGACCAG |
| 25G-TGA-F | | GACGGCGACGTAAACTGACACAAGTTCAGCGTG |
| 25G-TGA-R | | CACGCTGAACTTGTGTCAGTTTACGTCGCCGTC |
| 32G-TGA-F | | AAGTTCAGCGTGTCCTGAGAGGGCGAGGGCGAT |
| 32G-TGA-R | | ATCGCCCTCGCCCTCTCAGGACACGCTGAACTT |
| 34G-TGA-F | | AGCGTGTCCGGCGAGTGAGAGGGCGATGCCACC |
| 34G-TGA-R | | GGTGGCATCGCCCTCTCACTCGCCGGACACGCT |
| 41G-TGA-F | | GGCGATGCCACCTACTGAAAGCTGACCCTGAAG |
| 41G-TGA-R | | CTTCAGGGTCAGCTTTCAGTAGGTGGCATCGCC |
| 52G-TGA-F | | TTCATCTGCACCACCTGAAAGCTGCCCGTGCCC |
| 52G-TGA-R | | GGGCACGGGCAGCTTTCAGGTGGTGCAGATGAA |
| 58W-TGA-F | | AAGCTGCCCGTGCCCTGACCCACCCTCGTGACC |
| 58W-TGA-R | | GGTCACGAGGGTGGGTCAGGGCACGGGCAGCTT |
| 68G-TGA-F | | ACCACCCTGACCTACTGAGTGCAGTGCTTCAGC |
| 68G-TGA-R | | GCTGAAGCACTGCACTCAGTAGGTCAGGGTGGT |
| 74R-TGA-F | | GTGCAGTGCTTCAGCTGATACCCCGACCACATG |
| 74R-TGA-R | | CATGTGGTCGGGGTATCAGCTGAAGCACTGCAC |
| 92G-TGA-F | | TCCGCCATGCCCGAATGATACGTCCAGGAGCGC |
| 92G-TGA-R | | GCGCTCCTGGACGTATCATTCGGGCATGGCGGA |
| 105G-TGA-F | | TTCTTCAAGGACGACTGAAACTACAAGACCCG |
| 105G-TGA-R | | CGGGTCTTGTAGTTTCAGTCGTCCTTGAAGAA |
| 110R-TGA-F | | GGCAACTACAAGACCTGAGCCGAGGTGAAGTTC |
| 110R-TGA-R | | GAACTTCACCTCGGCTCAGGTCTTGTAGTTGCC |
| 117G-TGA-F | | GAGGTGAAGTTCGAGTGAGACACCCTGGTGAAC |
| 117G-TGA-R | | GTTCACCAGGGTGTCTCACTCGAACTTCACCTC |
| 123R-TGA-F | | GACACCCTGGTGAACTGAATCGAGCTGAAGGGC |
| 123R-TGA-R | | GCCCTTCAGCTCGATTCAGTTCACCAGGGTGTC |
| 128G-TGA-F | | CGCATCGAGCTGAAGTGAATCGACTTCAAGGAG |
| 128G-TGA-R | | CTCCTTGAAGTCGATTCACTTCAGCTCGATGCG |
| 135G-TGA-F | | GACTTCAAGGAGGACTGAAACATCCTGGGGCAC |
| 135G-TGA-R | | GTGCCCCAGGATGTTTCAGTCCTCCTTGAAGTC |
| 169R-TGA-F | | GTGAACTTCAAGATCTGACACAACATCGAGGAC |
| 169R-TGA-R | | GTCCTCGATGTTGTGTCAGATCTTGAAGTTCAC |
| 175G-TGA-F | | CACAACATCGAGGACTGAAGCGTGCAGCTCGCC |
| 175G-TGA-R | | GGCGAGCTGCACGCTTCAGTCCTCGATGTTGTG |
| 190G-TGA-F | | CAGAACACCCCCATCTGAGACGGCCCCGTGCTG |
| 190G-TGA-R | | CAGCACGGGGCCGTCTCAGATGGGGGTGTTCTG |
| 4K-TGA-F | | ATGGTGAGCTGAGGCGAGGAGCTGTTC |
| 4K-TGA-R | | GAACAGCTCCTCGCCTCAGCTCACCAT |
| 27K-TGA-F | | CGTAAACGGCCACTGATTCAGCGTGTCCG |
| 27K-TGA-R | | CGGACACGCTGAATCAGTGGCCGTTTACG |
| 42K-TGA-F | | ATGCCACCTACGGCTGACTGACCCTGAAG |
| 42K-TGA-R | | CTTCAGGGTCAGTCAGCCGTAGGTGGCAT |
| 53K-TGA-F | | CTGCACCACCGGCTGACTGCCCGTGCCCT |
| 53K-TGA-R | | AGGGCACGGGCAGTCAGCCGGTGGTGCAG |
| 80K-TGA-F | | CCCCGACCACATGTGACAGCACGACTTCTT |
| 80K-TGA-R | | AAGAAGTCGTGCTGTCACATGTGGTCGGGG |
| 86K-TGA-F | | GCACGACTTCTTCTGATCCGCCATGCCCG |
| 86K-TGA-R | | CGGGCATGGCGGATCAGAAGAAGTCGTGC |
| 114K-TGA-F | | CCGCGCCGAGGTGTGATTCGAGGGCGACAC |
| 114K-TGA-R | | GTGTCGCCCTCGAATCACACCTCGGCGCGG |
| 127K-TGA-F | | CCGCATCGAGCTGTGAGGCATCGACTTCAAG |
| 127K-TGA-R | | CTTGAAGTCGATGCCTCACAGCTCGATGCGG |
| 132K-TGA-F | | GGGCATCGACTTCTGAGAGGACGGCAACATC |
| 132K-TGA-R | | GATGTTGCCGTCCTCTCAGAAGTCGATGCCC |
| 159K-TGA-F | | GGCCGACAAGCAGTGAAACGGCATCAAGG |
| 159K-TGA-R | | CCTTGATGCCGTTTCACTGCTTGTCGGCC |
| 163K-TGA-F | | GAAGAACGGCATCTGAGTGAACTTCAAGA |
| 163K-TGA-R | | TCTTGAAGTTCACTCAGATGCCGTTCTTC |
| 167K-TGA-F | | TCAAGGTGAACTTCTGAATCCGCCACAACAT |
| 167K-TGA-R | | ATGTTGTGGCGGATTCAGAAGTTCACCTTGA |

**Table S2. Primers for construction of TAG harboring pDC-315-GFP mutants**

| Primer | Primer Sequence 5’→3’ | |
| --- | --- | --- |
| 315-GFP-5G-TAG-F | ATGGTGAGCAAGTAGGAGGAGCTGTTCACC | |
| 315-GFP-5G-TAG-R | GGTGAACAGCTCCTCCTACTTGCTCACCAT | |
| 315-GFP-11G-TAG-F | GAGGAGCTGTTCACCTAGGTGGTGCCCATCCTG | |
| 315-GFP-11G-TAG-R | CAGGATGGGCACCACCTAGGTGAACAGCTCCTC | |
| 315-GFP-21G-TAG-F | CTGGTCGAGCTGGACTAGGACGTAAACGGCCAC | |
| 315-GFP-21G-TAG-R | GTGGCCGTTTACGTCCTAGTCCAGCTCGACCAG | |
| 315-GFP-25G-TAG-F | GACGGCGACGTAAACTAGCACAAGTTCAGCGTG | |
| 315-GFP-25G-TAG-R | CACGCTGAACTTGTGCTAGTTTACGTCGCCGTC | |
| 315-GFP-32G-TAG-F | AAGTTCAGCGTGTCCTAGGAGGGCGAGGGCGAT | |
| 315-GFP-32G-TAG-R | ATCGCCCTCGCCCTCCTAGGACACGCTGAACTT | |
| 315-GFP-34G-TAG-F | AGCGTGTCCGGCGAGTAGGAGGGCGATGCCACC | |
| 315-GFP-34G-TAG-R | GGTGGCATCGCCCTCCTACTCGCCGGACACGCT | |
| 315-GFP-58W-TAG-F | AAGCTGCCCGTGCCCTAGCCCACCCTCGTGACC | |
| 315-GFP-58W-TAG-R | GGTCACGAGGGTGGGCTAGGGCACGGGCAGCTT | |
| 315-GFP-68G-TAG-F | ACCACCCTGACCTACTAGGTGCAGTGCTTCAGC | |
| 315-GFP-68G-TAG-R | GCTGAAGCACTGCACCTAGTAGGTCAGGGTGGT | |
| 315-GFP-74R-TAG-F | GTGCAGTGCTTCAGCTAGTACCCCGACCACATG | |
| 315-GFP-74R-TAG-R | CATGTGGTCGGGGTACTAGCTGAAGCACTGCAC | |
| 315-GFP-92G-TAG-F | TCCGCCATGCCCGAATAGTACGTCCAGGAGCGC | |
| 315-GFP-92G-TAG-R | GCGCTCCTGGACGTATCATTCGGGCATGGCGGA | |
| 315-GFP-105G-TAG-F | TTCTTCAAGGACGACTAGAACTACAAGACCCG | |
| 315-GFP-105G-TGA-R | CGGGTCTTGTAGTTCTAGTCGTCCTTGAAGAA | |
| 315-GFP-110R-TAG-F | GGCAACTACAAGACCTAGGCCGAGGTGAAGTTC | |
| 315-GFP-110R-TAG-R | GAACTTCACCTCGGCCTAGGTCTTGTAGTTGCC | |
| 315-GFP-117G-TAG-F | GAGGTGAAGTTCGAGTAGGACACCCTGGTGAAC | |
| 315-GFP-117G-TAG-R | GTTCACCAGGGTGTCCTACTCGAACTTCACCTC | |
| 315-GFP-123R-TAG-F | GACACCCTGGTGAACTAGATCGAGCTGAAGGGC | |
| 315-GFP-123R-TAG-R | GCCCTTCAGCTCGATCTAGTTCACCAGGGTGTC | |
| 315-GFP-128G-TAG-F | CGCATCGAGCTGAAGTAGATCGACTTCAAGGAG | |
| 315-GFP-128G-TAG-R | CTCCTTGAAGTCGATCTACTTCAGCTCGATGCG | |
| 315-GFP-135G-TAG-F | GACTTCAAGGAGGACTAGAACATCCTGGGGCAC | |
| 315-GFP-135G-TAG-R | GTGCCCCAGGATGTTCTAGTCCTCCTTGAAGTC | |
| 315-GFP-169R-TAG-F | GTGAACTTCAAGATCTAGCACAACATCGAGGAC | |
| 315-GFP-169R-TAG-R | GTCCTCGATGTTGTGCTAGATCTTGAAGTTCAC | |
| 315-GFP-175G-TAG-F | CACAACATCGAGGACTAGAGCGTGCAGCTCGCC | |
| 315-GFP-175G-TAG-R | GGCGAGCTGCACGCTCTAGTCCTCGATGTTGTG | |
| 315-GFP-190G-TAG-F | CAGAACACCCCCATCTAGGACGGCCCCGTGCTG | |
| 315-GFP-190G-TAG-R | CAGCACGGGGCCGTCCTAGATGGGGGTGTTCTG | |
| 315-GFP-4K-TAG-F | ATGGTGAGCTAGGGCGAGGAGCTGTTC | |
| 315-GFP-4K-TAG-R | GAACAGCTCCTCGCCCTAGCTCACCAT | |
| 315-GFP-27K-TAG-F | CGTAAACGGCCACTAGTTCAGCGTGTCCG | |
| 315-GFP-27K-TAG-R | CGGACACGCTGAACTAGTGGCCGTTTACG | |
| 315-GFP-42K-TAG-F | ATGCCACCTACGGCTAGCTGACCCTGAAG | |
| 315-GFP-42K-TAG-R | CTTCAGGGTCAGCTAGCCGTAGGTGGCAT | |
| 315-GFP-53K-TAG-F | CTGCACCACCGGCTAGCTGCCCGTGCCCT | |
| 315-GFP-53K-TAG-R | AGGGCACGGGCAGCTAGCCGGTGGTGCAG | |
| 315-GFP-80K-TAG-F | CCCCGACCACATGTAGCAGCACGACTTCTT | |
| 315-GFP-80K-TAG-R | AAGAAGTCGTGCTGCTACATGTGGTCGGGG | |
| 315-GFP-86K-TAG-F | GCACGACTTCTTCTAGTCCGCCATGCCCG | |
| 315-GFP-86K-TAG-R | CGGGCATGGCGGACTAGAAGAAGTCGTGC | |
| 315-GFP-114K-TAG-F | CCGCGCCGAGGTGTAGTTCGAGGGCGACAC | |
| 315-GFP-114K-TAG-R | GTGTCGCCCTCGAACTACACCTCGGCGCGG | |
| 315-GFP-127K-TAG-F | CCGCATCGAGCTGTAGGGCATCGACTTCAAG | |
| 315-GFP-127K-TAG-R | CTTGAAGTCGATGCCCTACAGCTCGATGCGG | |
| 315-GFP-132K-TAG-F | GGGCATCGACTTCTAGGAGGACGGCAACATC | |
| 315-GFP-132K-TAG-R | GATGTTGCCGTCCTCCTAGAAGTCGATGCCC | |
| 315-GFP-159K-TAG-F | GGCCGACAAGCAGTAGAACGGCATCAAGG | |
| 315-GFP-159K-TAG-R | CCTTGATGCCGTTCTACTGCTTGTCGGCC | |
| 315-GFP-163K-TAG-F | GAAGAACGGCATCTAGGTGAACTTCAAGA | |
| 315-GFP-163K-TAG-R | TCTTGAAGTTCACCTAGATGCCGTTCTTC | |
| 315-GFP-167K-TAG-F | TCAAGGTGAACTTCTAGATCCGCCACAACAT | |
| 315-GFP-167K-TAG-R | ATGTTGTGGCGGATCTAGAAGTTCACCTTGA | |
|  |  |  |

**Table S3. Oligonucleotides for construction of single and double PTC of gag gene.**

| Primer | Primer Sequence 5’→3’ | |
| --- | --- | --- |
| GAG221G-F | | CATGACCTATTGCACCAGGCCAGATGAGAGAACCAAGGGGAAGTGACATAGCAGGAACTA |
| GAG221G-R | | CTAGTAGTTCCTGCTATGTCACTTCCCCTTGGTTCTCTCATCTGGCCTGGTGCAATAGGTCATGCATG |
| GAG226G-F | | CAGGGCCTATTGCACCATGACAGATGAGAGAACCAAGGGGAAGTGACATAGCAGGAACTA |
| GAG226G-R | | CTAGTAGTTCCTGCTATGTCACTTCCCCTTGGTTCTCTCATCTGTCATGGTGCAATAGGCCCTGCATG |
| GAG233G-F | | CAGGGCCTATTGCACCAGGCCAGATGAGAGAACCAAGGTGAAGTGACATAGCAGGAACTA |
| GAG233G-R | | CTAGTAGTTCCTGCTATGTCACTTCACCTTGGTTCTCTCATCTGGCCTGGTGCAATAGGCCCTGCATG |
| GAG238G-F | | CAGGGCCTATTGCACCAGGCCAGATGAGAGAACCAAGGGGAAGTGACATAGCATGAACTA |
| GAG238G-R | | CTAGTAGTTCATGCTATGTCACTTCCCCTTGGTTCTCTCATCTGGCCTGGTGCAATAGGCCCTGCATG |
| GAG229R-F | | CAGGGCCTATTGCACCAGGCCAGATGTGAGAACCAAGGGGAAGTGACATAGCAGGAACTA |
| GAG229R-R | | CTAGTAGTTCCTGCTATGTCACTTCCCCTTGGTTCTCACATCTGGCCTGGTGCAATAGGCCCTGCATG |
| GAG232R-F | | CAGGGCCTATTGCACCAGGCCAGATGAGAGAACCATGAGGAAGTGACATAGCAGGAACTA |
| GAG232R-R | | CTAGTAGTTCCTGCTATGTCACTTCCTCATGGTTCTCTCATCTGGCCTGGTGCAATAGGCCCTGCATG |
| GAG221G/226G-F | | CATGACCTATTGCACCATGACAGATGAGAGAACCAAGGGGAAGTGACATAGCAGGAACTA |
| GAG221G/226G-R | | CTAGTAGTTCCTGCTATGTCACTTCCCCTTGGTTCTCTCATCTGTCATGGTGCAATAGGTCATGCATG |
| GAG221G/238G-F | | CATGACCTATTGCACCAGGCCAGATGAGAGAACCAAGGGGAAGTGACATAGCATGAACTA |
| GAG221G/238G-R | | CTAGTAGTTCATGCTATGTCACTTCCCCTTGGTTCTCTCATCTGGCCTGGTGCAATAGGTCATGCATG |
| GAG229R/232R-F | | CAGGGCCTATTGCACCAGGCCAGATGTGAGAACCATGAGGAAGTGACATAGCAGGAACTA |
| GAG229R/232R-R | | CTAGTAGTTCCTGCTATGTCACTTCCTCATGGTTCTCACATCTGGCCTGGTGCAATAGGCCCTGCATG |

**Table S4. Primers for construction of PTC harboring gB mutants.**

| Primer | Primer Sequence 5’→3’ |
| --- | --- |
| gB-9R-TGA-F | GgtggcggtctttggTGAgggccccgcgggcat |
| gB-9R-TGA-R | atgcccgcggggcccTCAccaaagaccgccacc |
| gB-26R-TGA-F | ggtgctggcctcggaTGActttggcctgctcca |
| gB-26R-TGA-R | tggagcaggccaaagTCAtccgaggccagcacc |
| gB-111R-TGA-F | ggcgacctggacgcgTGAacggccgtgcgcgcg |
| gB-111R-TGA-R | cgcgcgcacggccgtTCAcgcgtccaggtcgcc |
| gB-115R-TGA-F | gcgcgcacggccgtgTGAgcggccgcgaccgag |
| gB-115R-TGA-R | ctcggtcgcggccgcTCAcacggccgtgcgcgc |
| gB-123R-TGA-F | gcgaccgagcgggacTGAttctacgtctgcccg |
| gB-123R-TGA-R | cgggcagacgtagaaTCAgtcccgctcggtcgc |
| gB-137R-TGA-F | ggctccacggtggtgTGActggagcccgagcag |
| gB-137R-TGA-R | ctgctcgggctccagTCAcaccaccgtggagcc |
| gB-151R-TGA-F | gagtactcgcaggggTGAaacttcacggagggg |
| gB-151R-TGA-R | cccctccgtgaagttTCAcccctgcgagtactc |
| gB-196R-TGA-F | gcggccatcacgaacTGAttcacggaccgcgtg |
| gB-196R-TGA-R | cacgcggtccgtgaaTCAgttcgtgatggccgc |
| gB-226R-TGA-F | aaggccgagtacgtgTGAaacaaccacaaggtg |
| gB-226R-TGA-R | caccttgtggttgttTCAcacgtactcggcctt |
| gB-246R-TGA-F | gtcgaggtggacctgTGAccctcgcgcctgaac |
| gB-246R-TGA-R | gttcaggcgcgagggTCAcaggtccacctcgac |
| gB-256R-TGA-F | aacgcgctcggcaccTGAggctggcacaccacc |
| gB-256R-TGA-R | ggtggtgtgccagccTCAggtgccgagcgcgtt |
| gB-290R-TGA-F | gaggaggtggaggcgTGAtccgtgtacccctac |
| gB-290R-TGA-R | gtaggggtacacggaTCAcgcctccacctcctc |
| gB-315R-TGA-F | cccttctacggcctgTGAgagggggcccacggg |
| gB-315R-TGA-R | cccgtgggccccctcTCAcaggccgtagaaggg |
| gB-329R-TGA-F | ggctacgcgcccgggTGAttccagcaggtggag |
| gB-329R-TGA-R | ctccacctgctggaaTCAcccgggcgcgtagcc |
| gB-357R-TGA-F | acgcgcaactttctgTGAacgccgcacttcacg |
| gB-357R-TGA-R | cgtgaagtgcggcgtTCAcagaaagttgcgcgt |
| gB-17G-TGA-F | cgggcatcggcccTGAcaccacggcggtgctgg |
| gB-17G-TGA-R | ccagcaccgccgtggtgTCAgggccgatgcccg |
| gB-39G-TGA-F | gctgcagctgcgcggTGAgccgtcgcgctagcg |
| gB-39G-TGA-R | cgctagcgcgacggcTCAccgcgcagctgcagc |
| gB-57G-TGA-F | gcgaccccgacgtgcTGAgcggcggccgtgacg |
| gB-57G-TGA -R | cgtcacggccgccgcTCAgcacgtcggggtcgc |
| gB-79G-TGA-F | ggcgccaccccagacTGAttctccgcggaggag |
| gB-79G-TGA-R | ctcctccgcggagaaTCAgtctggggtggcgcc |
| gB-91G-TGA-F | ctcgaggagatcgacTGAgccgtctcccccggc |
| gB-91G-TGA-R | gccgggggagacggcTCAgtcgatctcctcgag |
| gB-132G-TGA-F | tgcccgccgccgtccTGAtccacggtggtgcgc |
| gB-132G-TGA-R | gcgcaccaccgtggaTCAggacggcggcgggca |
| gB-187G-TGA-F: | acgaccgtgtggtccTGAagcacgtacgcggcc |
| gB-187G-TGA-R: | ggccgcgtacgtgctTCAggaccacacggtcgt |
| gB-216G-TGA-F: | gtgatcgaccgccgcTGAaagtgcgtctccaag |
| gB-216G-TGA-R: | cttggagacgcacttTCAgcggcggtcgatcac |
| gB-254G-TGA-F: | cgcctgaacgcgctcTGAacccgcggctggcac |
| gB-254G-TGA-R: | gtgccagccgcgggtTCAgagcgcgttcaggcg |
| gB-303G-TGA-F: | ttcgccctgtccacgTGAgacatcgtgtacatg |
| gB-303G-TGA-R: | catgtacacgatgtcTCAcgtggacagggcgaa |
| gB-328G-TGA-F: | atcggctacgcgcccTGAcgcttccagcaggtg |
| gB-328G-TGA-R: | cacctgctggaagcgTCAgggcgcgtagccgat |
| gB-394G-TGA-F: | gacgagacgcgcgacTGAtccttccgcttcacg |
| gB-394G-TGA-R: | cgtgaagcggaaggaTCAgtcgcgcgtctcgtc |
| gB-404G-TGA-F: | acgtcgcgggccctgTGAgcctccttcgtcagc |
| gB-404G-TGA-R: | gctgacgaaggaggcTCAcagggcccgcgacgt |
| gB-422G-TGA-F: | cagcgcgtgcacctgTGAgactgcgtcctccgc |
| gB-422G-TGA-R: | gcggaggacgcagtcTCAcaggtgcacgcgctg |

**Table S5.** Primers and probes used for Real-time PCR.

| Primer | Primer Sequence 5’→3’ |
| --- | --- |
| 315GFP-probe | ACATGGTCCTGCTGGAGTTCGTGACCG |
| 315GFP-F | AAAGACCCCAACGAGAAGCG |
| 315GFP-R | TCGTCCATGCCGAGAGTGA |
| HIV-Gag-probe | ACCAGAGCCAACAGCCCCACCAGAAGA |
| HIV-Gag-F | GGAAGATCTGGCCTTCCCACA |
| HIV-Gag-R | CTCTTCCCCAAACCTGAAGCTC |
| gB-probe  gB-F  gB-R | CTCGCGCGACCTCATCGAGCCCTGCAC  ACGGCACGGGCGTGATC  ACTCGCGGTCCTCGAGCA |

1. Wang, T.; Tong, W.; Ye, C.; Yu, Z.; Chen, J.; Gao, F.; Shan, T.; Yu, H.; Li, L.; Li, G.; Tong, G.; Zheng, H., Construction of an infectious bacterial artificial chromosome clone of a pseudorabies virus variant: Reconstituted virus exhibited wild-type properties in vitro and in vivo. *J Virol Methods* **2018,** *259*, 106-115.

2. (a) Tang, Y. D.; Na, L.; Zhu, C. H.; Shen, N.; Yang, F.; Fu, X. Q.; Wang, Y. H.; Fu, L. H.; Wang, J. Y.; Lin, Y. Z.; Wang, X. F.; Wang, X.; Zhou, J. H.; Li, C. Y., Equine viperin restricts equine infectious anemia virus replication by inhibiting the production and/or release of viral Gag, Env, and receptor via distortion of the endoplasmic reticulum. *Journal of virology* **2014,** *88* (21), 12296-310; (b) Wang, Y.; Liu, T. X.; Wang, T. Y.; Tang, Y. D.; Wei, P., Isobavachalcone inhibits Pseudorabies virus by impairing virus-induced cell-to-cell fusion. *Virology journal* **2020,** *17* (1), 39.

3. Yang, Y. L.; Meng, F.; Qin, P.; Herrler, G.; Huang, Y. W.; Tang, Y. D., Trypsin promotes porcine deltacoronavirus mediating cell-to-cell fusion in a cell type-dependent manner. *Emerging microbes & infections* **2020,** *9* (1), 457-468.

4. Zhang, H. L.; Li, Y. M.; Sun, J.; Zhang, Y. Y.; Wang, T. Y.; Sun, M. X.; Wang, M. H.; Yang, Y. L.; Hu, X. L.; Tang, Y. D.; Zhao, J.; Cai, X., Evaluating angiotensin-converting enzyme 2-mediated SARS-CoV-2 entry across species. *The Journal of biological chemistry* **2021**, 100435.

5. (a) Klupp, B. G.; Nixdorf, R.; Mettenleiter, T. C., Pseudorabies virus glycoprotein M inhibits membrane fusion. *J Virol* **2000,** *74* (15), 6760-8; (b) Vallbracht, M.; Rehwaldt, S.; Klupp, B. G.; Mettenleiter, T. C.; Fuchs, W., Functional Relevance of the N-Terminal Domain of Pseudorabies Virus Envelope Glycoprotein H and Its Interaction with Glycoprotein L. *J Virol* **2017,** *91* (9).
